# Supplementary material for: Photothermally heated colloidal synthesis of nanoparticles driven by silica-encapsulated plasmonic heat sources
Source: Nat Commun. 2023 Oct 10;14:6355. doi: 10.1038/s41467-023-42167-9 (PMC10564728; doi:10.1038/s41467-023-42167-9)
Supplement: Supplementary file 1 — Supplementary Information [file 41467_2023_42167_MOESM1_ESM.pdf]

# Supplementary Information

## Photothermally heated colloidal synthesis of nanoparticles driven by silica-encapsulated plasmonic heat sources

Aritra Biswas[1], Nir Lemcoff[1], Ofir Shelonchik[1], Doron Yesodi[1], Elad Yehezkel[1], Ella Yonit Finestone[1], Alexander Upcher[2], and Yossi Weizmann*[1,2,3]

[1]Department of Chemistry, Ben-Gurion University of the Negev, Beer-Sheva 84105, Israel
[2]Ilse Katz Institute for Nanotechnology Science, Ben-Gurion University of the Negev, Beer-Sheva 84105, Israel
[3]Goldman Sonnenfeldt School of Sustainability and Climate Change, Ben-Gurion University of the Negev, Beer-Sheva 84105, Israel

*Correspondence to: yweizmann@bgu.ac.il (Y.W.)

## Table of Contents

1. Supplementary Figures 1-50. ....4

Supplementary Figure 1 ....4
Supplementary Figure 2 ....4
Supplementary Figure 3 ....5
Supplementary Figure 4 ....5
Supplementary Figure 5 ....6
Supplementary Figure 6 ....7
Supplementary Figure 7 ....7
Supplementary Figure 8 ....8
Supplementary Figure 9 ....9
Supplementary Figure 10 ....9
Supplementary Figure 11 ....10
Supplementary Figure 12 ....11
Supplementary Figure 13 ....12
Supplementary Figure 14 ....13
Supplementary Figure 15 ....14
Supplementary Figure 16 ....15
Supplementary Figure 17 ....16
Supplementary Figure 18 ....17
Supplementary Figure 19 ....17
Supplementary Figure 20 ....18
Supplementary Figure 21 ....18
Supplementary Figure 22 ....19
Supplementary Figure 23 ....19
Supplementary Figure 24 ....20
Supplementary Figure 25 ....21
Supplementary Figure 26 ....22
Supplementary Figure 27 ....23
Supplementary Figure 28 ....23
Supplementary Figure 29 ....24
Supplementary Figure 30 ....24
Supplementary Figure 31 ....25
Supplementary Figure 32 ....26
Supplementary Figure 33 ....27
Supplementary Figure 34 ....27
Supplementary Figure 35 ....28

Supplementary Figure 36 ........................................................................................................28
Supplementary Figure 37 ........................................................................................................29
Supplementary Figure 38 ........................................................................................................29
Supplementary Figure 39 ........................................................................................................30
Supplementary Figure 40 ........................................................................................................30
Supplementary Figure 41 ........................................................................................................31
Supplementary Figure 42 ........................................................................................................32
Supplementary Figure 43 ........................................................................................................33
Supplementary Figure 44 ........................................................................................................33
Supplementary Figure 45 ........................................................................................................34
Supplementary Figure 46 ........................................................................................................35
Supplementary Figure 47 ........................................................................................................36
Supplementary Figure 48 ........................................................................................................36
Supplementary Figure 49 ........................................................................................................37
Supplementary Figure 50 ........................................................................................................37
2. Supplementary Tables 1-6. ....................................................................................................38
Supplementary Table 1 ............................................................................................................38
Supplementary Table 2 ............................................................................................................38
Supplementary Table 3 ............................................................................................................38
Supplementary Table 4 ............................................................................................................38
Supplementary Table 5 ............................................................................................................39
Supplementary Table 6 ............................................................................................................39

## 1. Supplementary Figures 1-50.

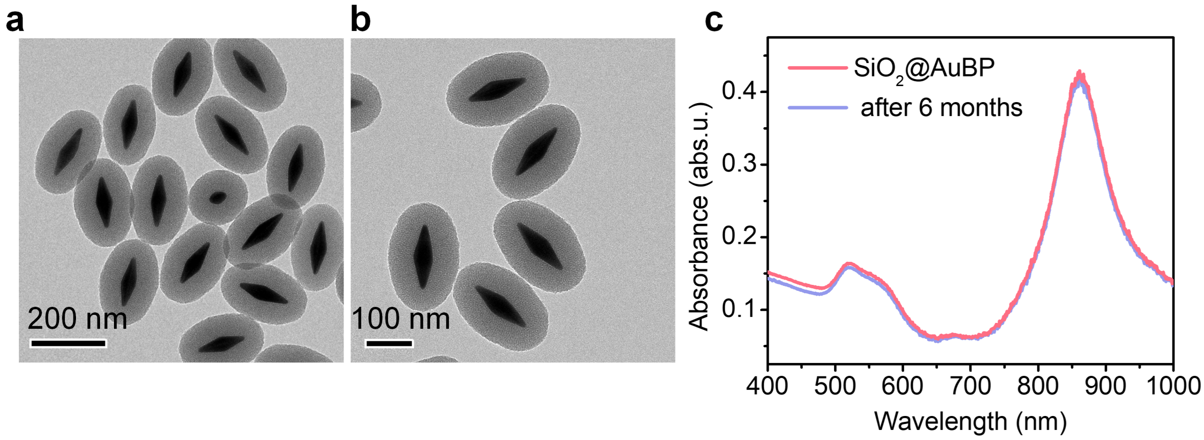


**Supplementary Figure 1 | Transmission electron microscope (TEM) image, and UV-Vis absorption spectrum.** TEM images of silica encapsulated $AuBP_{850}$, **a,** before, and **b,** after more than six months of storage under 4 °C in ethanol, exhibiting excellent colloidal stability as observed from the **c,** UV-Vis spectrum. Source data are provided as a Source Data file.

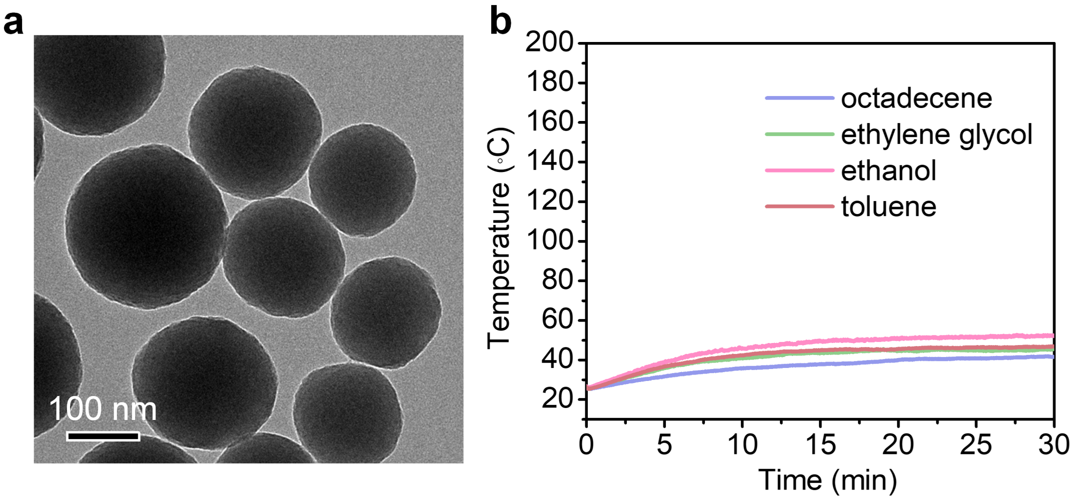


**Supplementary Figure 2 | a,** TEM image of silica nanoparticles with size ≈150-200 nm and **b,** temperature profile in presence of silica nanoparticles (1mg $mL^{-1}$) in different solvents demonstrating insignificant contribution to the photothermal temperature under 850 nm light irradiation. Source data are provided as a Source Data file.

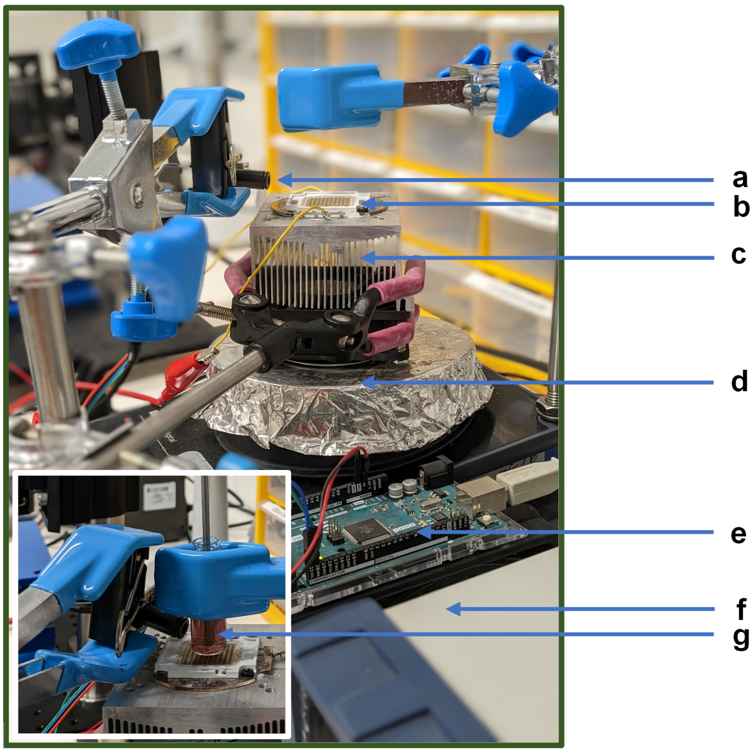


**Supplementary Figure 3 | Digital image of photothermal setup with an IR thermometer sensor connected to the computer by an Arduino Mega for nanoparticle synthesis. a,** MLX90614-DF ROBOT IR thermometer sensor. **b,** LCFOCUS 100 W, 850 nm, output 6-7 W $cm^{-2}$. **c,** aluminum heat-sink with a radiator cooling fan. **d,** IKA plate for stirring the solution (temperature was set off). **e,** Arduino Mega. **f,** 9130B-BK. **g,** calibration of IR thermometer with a conventional thermometer by PT 1000.60 temperature sensor from a RCT digital IKA plate (inset). During a 10-minute reaction at 200 °C, the energy consumption (power in watts times the time period of the reaction in seconds) for conventional heating amounts to approximately 360 kJ, whereas for the photothermal reaction, it is notably lower, around 28 kJ.

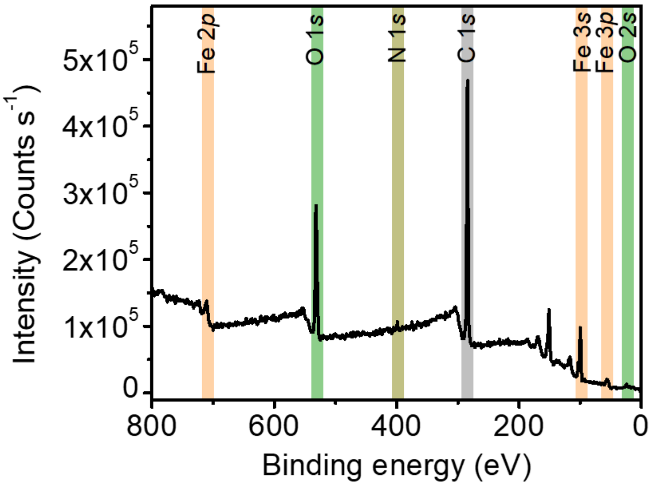


**Supplementary Figure 4 | Survey scan X-ray photoelectron spectroscopy (XPS).** Survey scan XPS reveals the presence of elemental Fe (orange), and O (green) as main elements in IONPs. Source data are provided as a Source Data file.

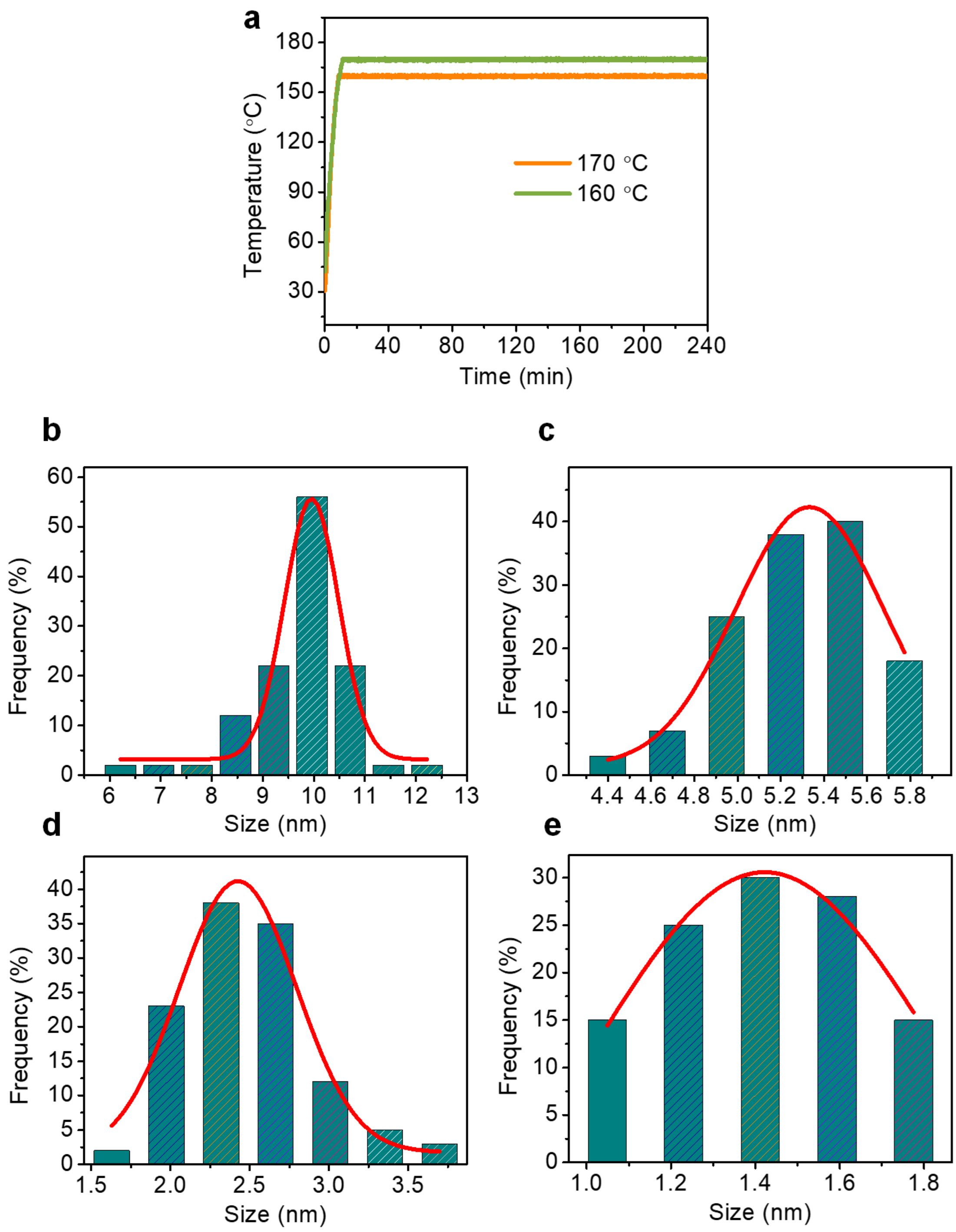


**Supplementary Figure 5 | Temperature profile and size distribution of IONPs synthesized at different photothermal temperatures. a,** Temperature profile for photothermal synthesis of IONPs at 160 and 170 °C. Size distributions of the synthesized nanoparticles at photothermal temperature of **b,** 200 °C (average size ≈10 nm, n=122, SD 0.9 nm), **c,** 180 °C (average size ≈5.3 nm, n=131, SD 0.4 nm), **d,** 170 °C (average size ≈2.4 nm, n=150, SD 0.2 nm), and **e,** 160 °C (average size ≈1.4 nm, n=113, SD 0.1 nm). SD: standard deviation. The distributions were obtained by analyzing ≥100 nanoparticles (n) chosen arbitrarily for each reaction with Image J software, and fitted (red curve) using gaussian function. Source data are provided as a Source Data file.

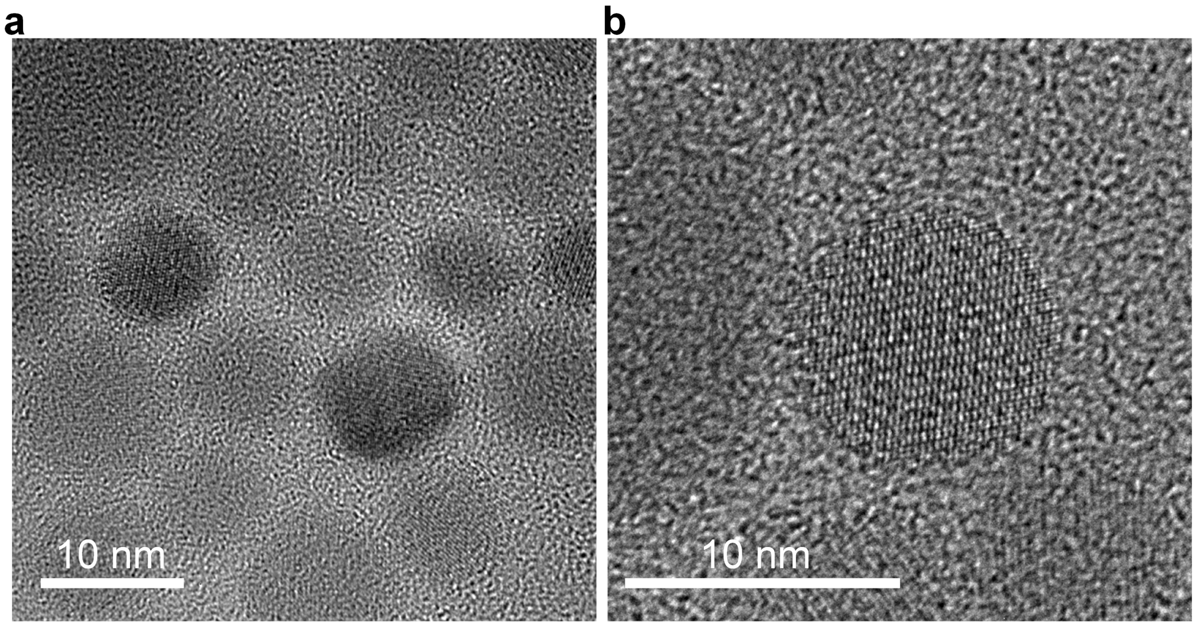


**Supplementary Figure 6** | **a-b**, High-resolution TEM (HR-TEM) images of photothermally synthesized ≈10 nm IONPs at different magnifications.

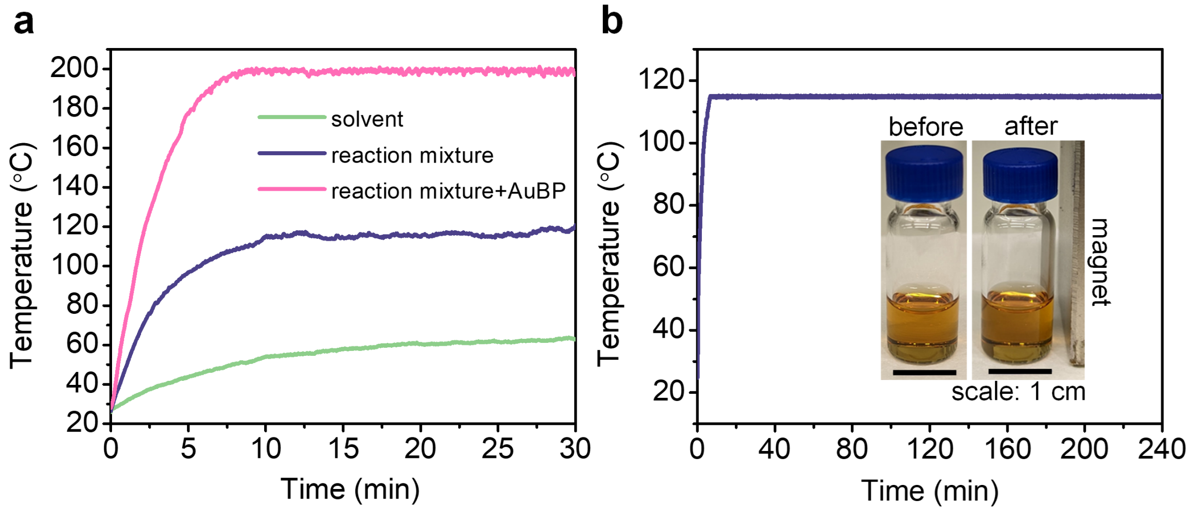


**Supplementary Figure 7** | **a,** Temperature profile of control reactions with and without AuBPs under the same photothermal condition indicating the leading role of AuBPs in the synthesis of iron oxide nanoparticles, and without AuBPs the overall temperature is much below the nucleation temperature of the nanoparticle. **b,** Temperature profile for reaction mixture without AuBPs which is photothermally heated for 4 h at its maximum capability highlighting no isolated nanoparticle formation. Inset showing reaction mixture before, and after the reaction without any nanoparticle formation. Source data are provided as a Source Data file.

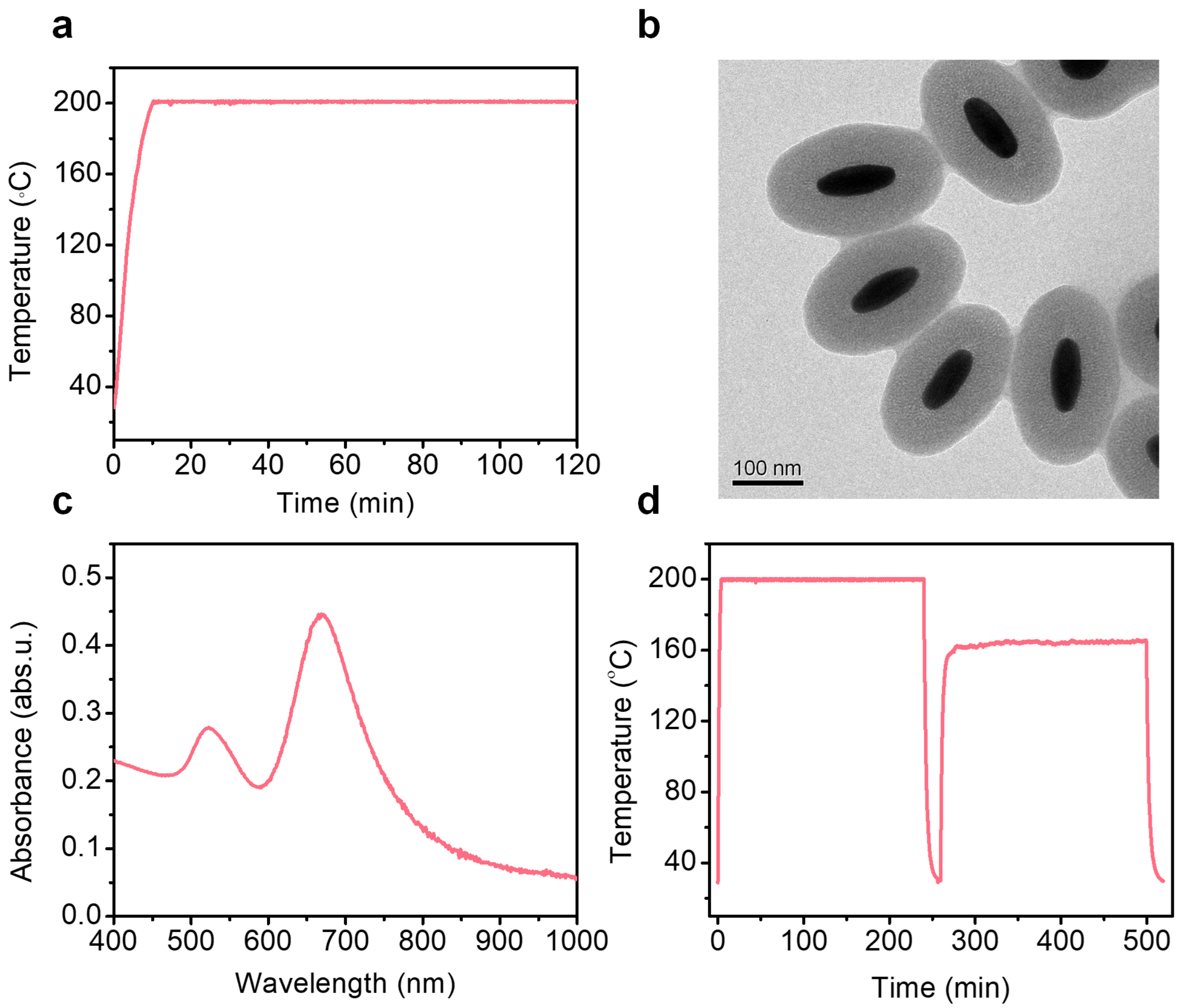


**Supplementary Figure 8** | **a,** Temperature profile of $SiO_2$@$AuBP_{850}$ in octadecence-oleic acid-oleylamine mixture without iron oleate under 850 nm light irradiation. **b,** TEM image after 60 min of reaction showing degradation of the sharp tips of AuBPs keeping overall structural stability intact. **c,** UV-Vis absorbance spectrum of the corresponding nanoparticles showing shifting off of the LSPR to a lower wavelength. **d,** Temperature profile of $SiO_2$@$AuBP_{850}$ with iron oleate in octadecence-oleic acid-oleylamine mixture followed at 200 °C for 4 h. Subsequently the LED was turned off, and the reaction mixture was cooled at room temperature. The LED under the same condition was turned on afterward, exhibiting the AuBP nanoparticles' heating ability slowed down and could reach only ≈160-165 °C. The temperature however remained consistently maintained high for 2-4 h throughout for both the reactions (a, d) due to weak absorption of AuBPs around 850 nm, and likely because once the set temperature programmed by LabVIEW is reached, despite the fact that the bipyramids undergo a gradual reshaping process, it's easier to maintain stability. Source data are provided as a Source Data file.

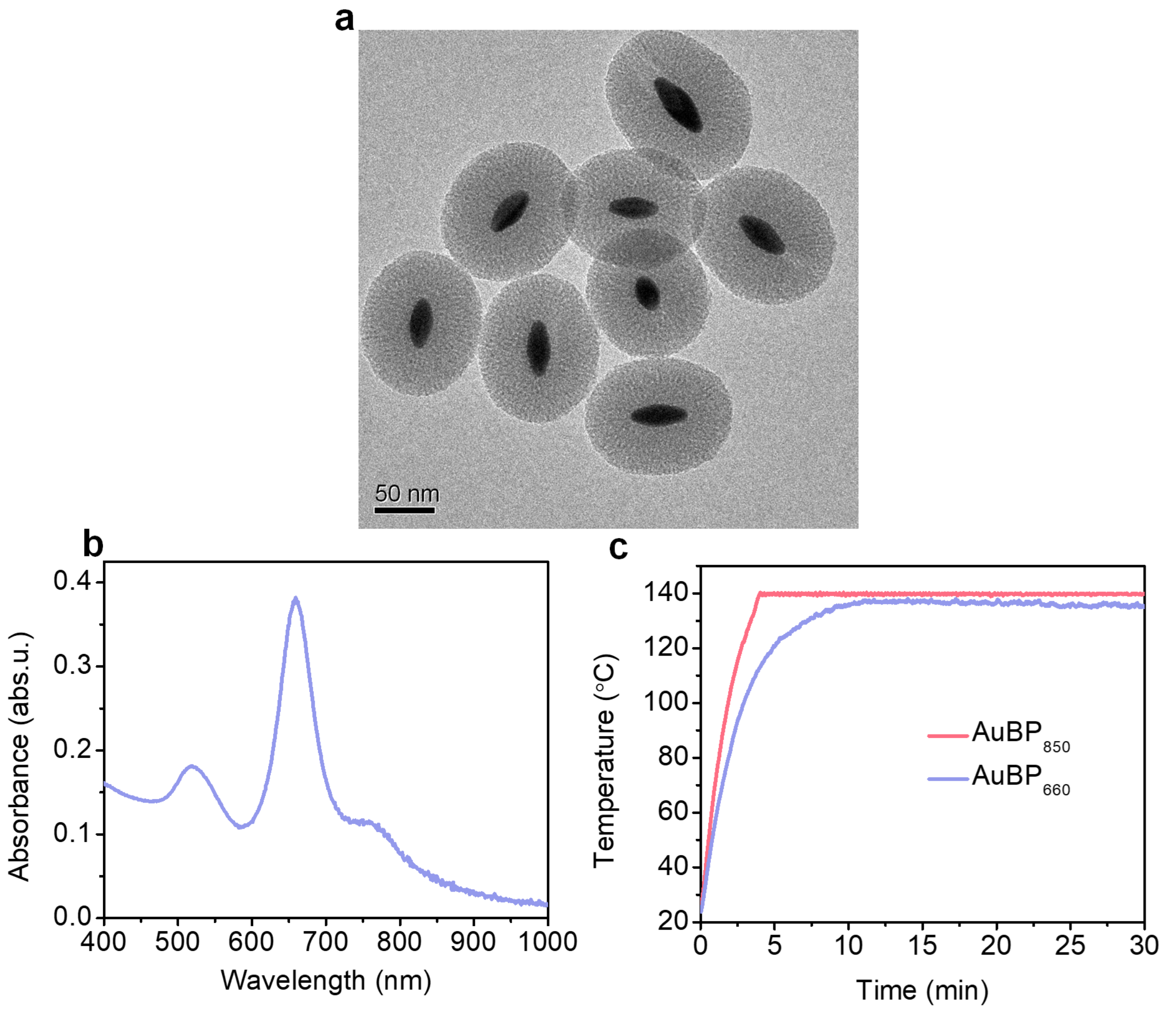


**Supplementary Figure 9** | **a,** TEM image of $SiO_2$@$AuBP_{660}$ with corresponding **b,** UV-Vis absorption spectrum. **c,** Temperature profile of silica-coated AuBPs in DMF under 850 nm light irradiation for 30 min demonstrating slow heating profile of $AuBP_{660}$ (4 OD measured at 660 nm) compared to $AuBP_{850}$ (4 OD measured at 850 nm) which failed to reach a set temperature of 140 °C. Source data are provided as a Source Data file.

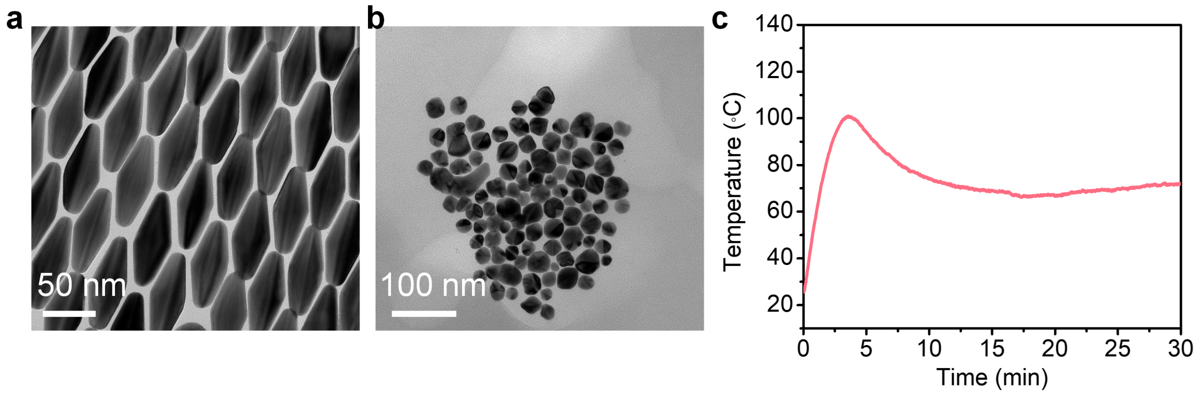


**Supplementary Figure 10** | **a,** TEM image of non-encapsulated $AuBP_{850}$ prior to photothermal activation. **b,** Images taken of the AuBPs after conducting the photothermal heating 4 OD

(measured at 850 nm) in DMF under 850 nm light irradiation, which failed to reach or maintain the set photothermal temperature of 140 °C under a similar condition as shown in Supplementary Fig. 9c. Source data are provided as a Source Data file.

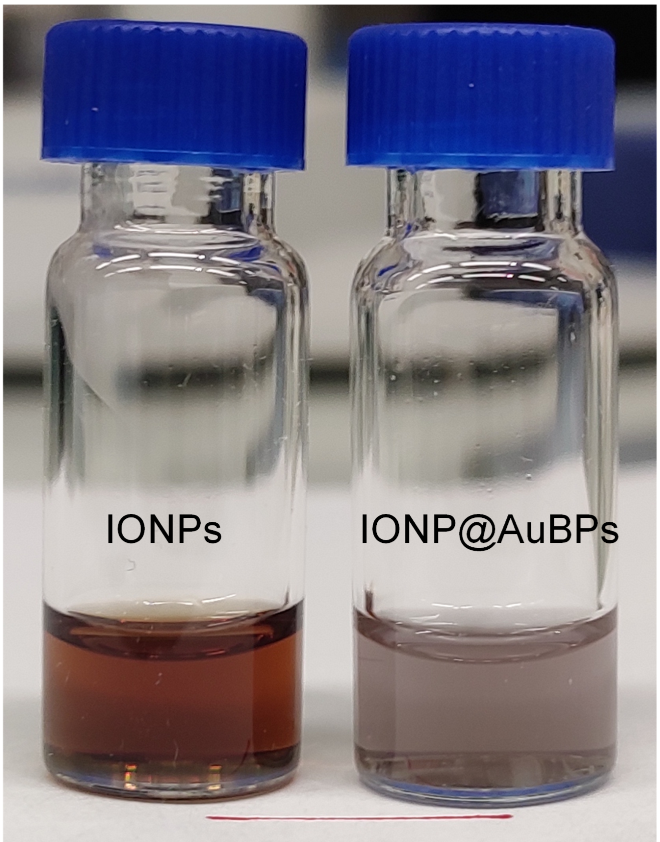


**Supplementary Figure 11** | Representative digital image showing the purified nanoparticles after the photothermal reaction at 180-200 °C, 2-3 h. Left, brown-black colored-colloidal solution of IONPs in toluene, and right, violet colored-colloidal solution of IONP@AuBPs in ethanol. Scale bar is 1 cm.

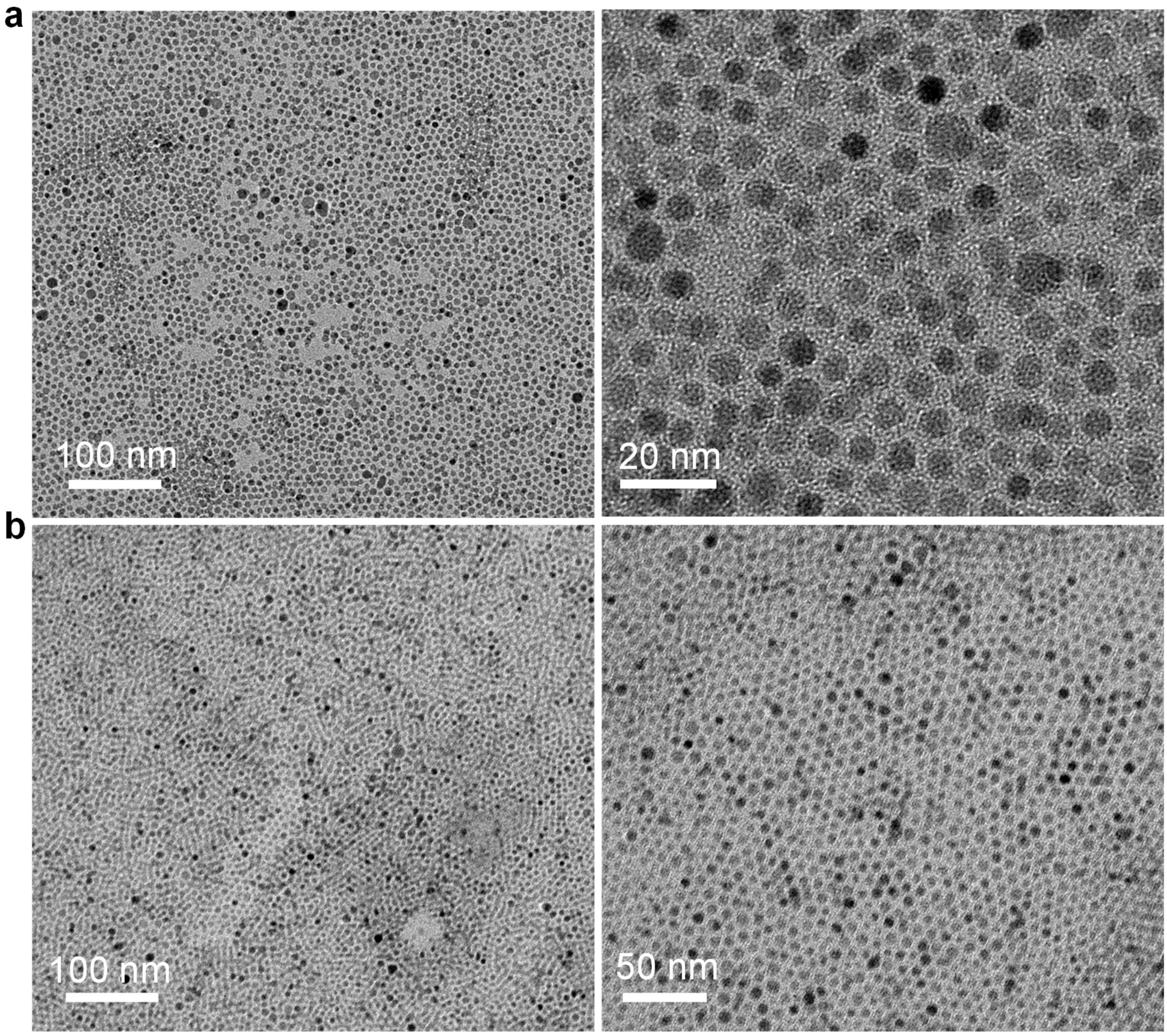


**Supplementary Figure 12** | Additional TEM images of IONPs at different magnifications synthesized photothermally at 200 °C, for **a,** 3 h, and **b,** 2 h.

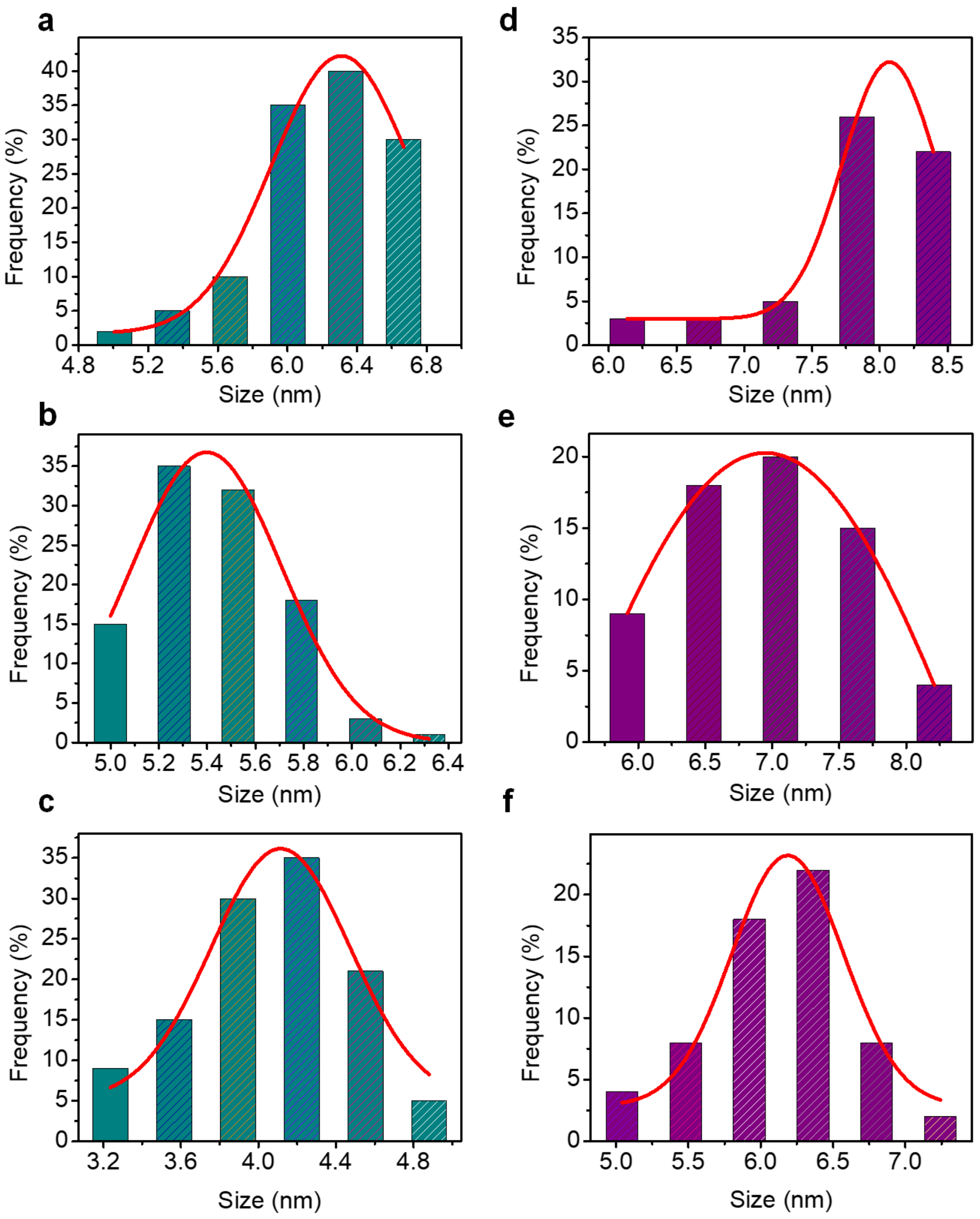


**Supplementary Figure 13** | Size distribution of IONPs (**a-c,** cyan), and IONPs on the AuBPs (**d-f,** magenta) synthesized at photothermal temperature of 200 °C at different time intervals. **a,** IONPs: ≈6.3 nm, n=122, SD 0.5 nm, **d,** IONPs on AuBP: ≈8.1 nm, n=59, SD 0.6 nm for 180 min reaction, **b,** IONPs: ≈5.4 nm, n=104, SD 0.4 nm, **e,** IONPs on AuBP: ≈7 nm, n=66, SD 0.6 nm for 120 min reaction, and **c,** IONPs: ≈4.1 nm, n=115, SD 0.3 nm, **f,** IONPs on AuBP: ≈6.2 nm, n=62, SD 0.5 nm for 45 min reaction. SD: standard deviation. The distributions were obtained by analyzing ≥100 IONPs, and ≥50 IONPs on the AuBPs (n) chosen arbitrarily for each reaction with ImageJ software, and fitted using gaussian function (red curve). Source data are provided as a Source Data file.

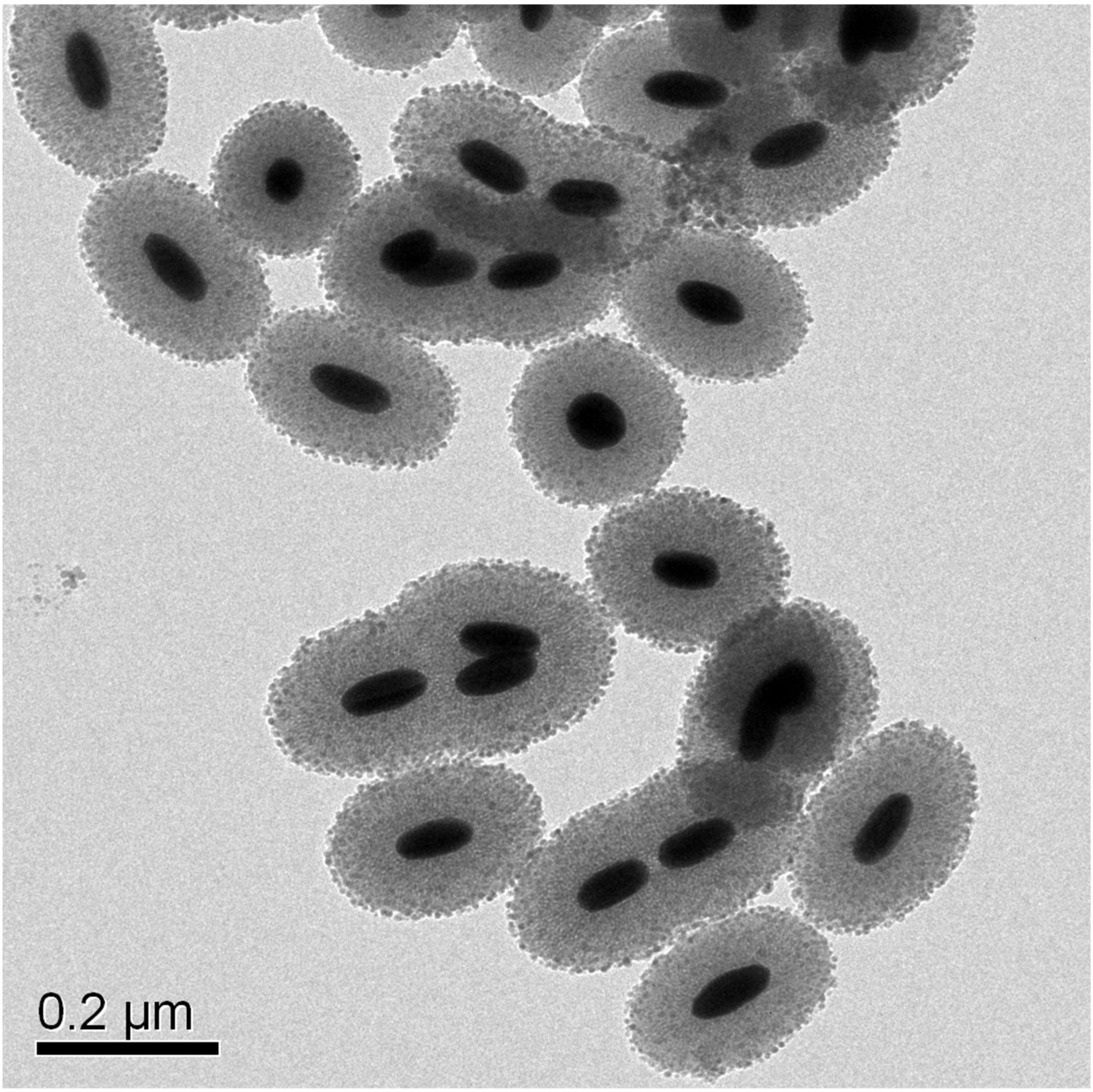


**Supplementary Figure 14** | Low magnification TEM image of IONP@AuBP nanohybrids photothermally synthesized at 200 °C for 45 min.

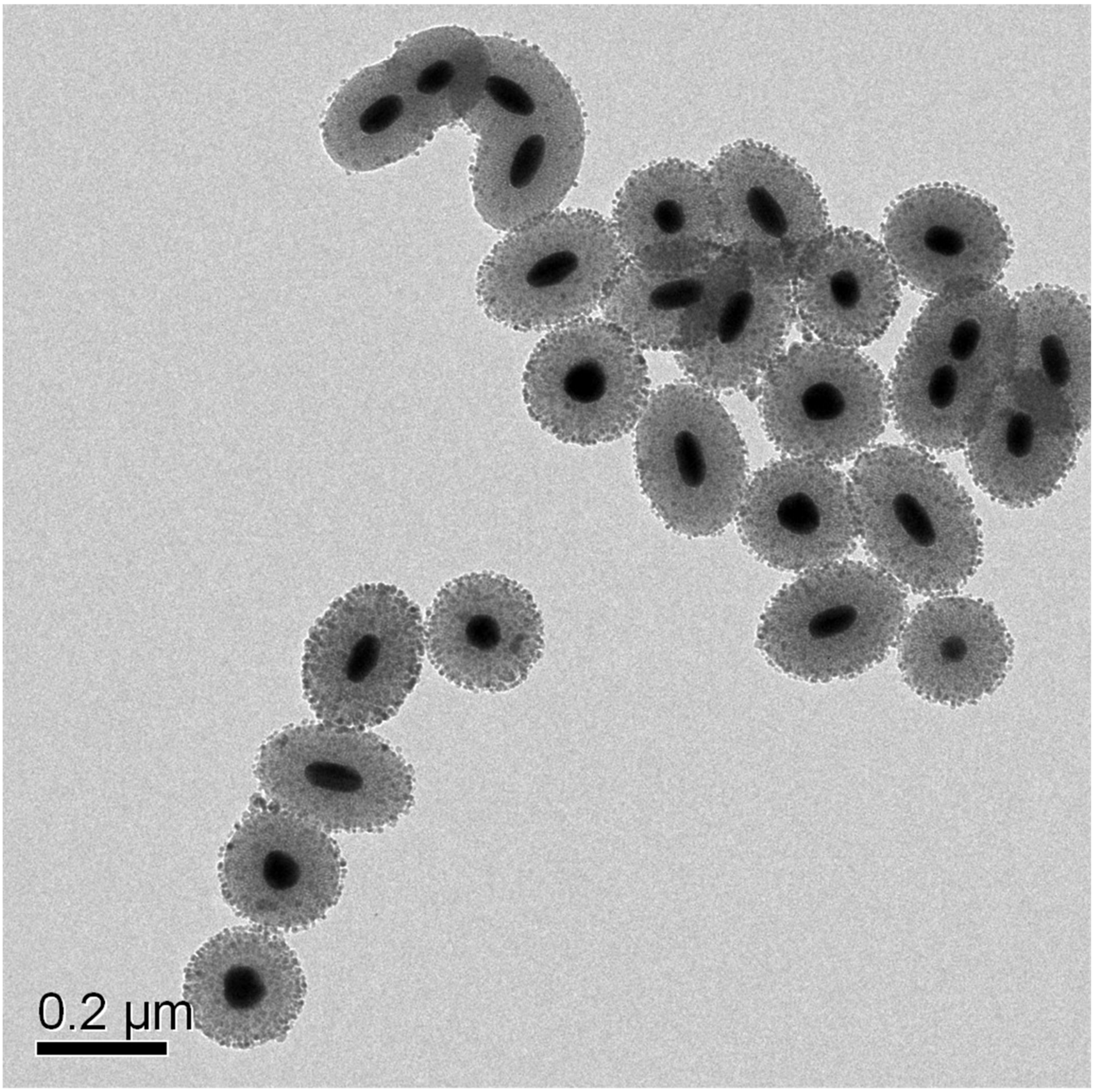


**Supplementary Figure 15** | Low magnification TEM image of IONP@AuBP nanohybrid photothermally synthesized at 200 °C for 120 min.

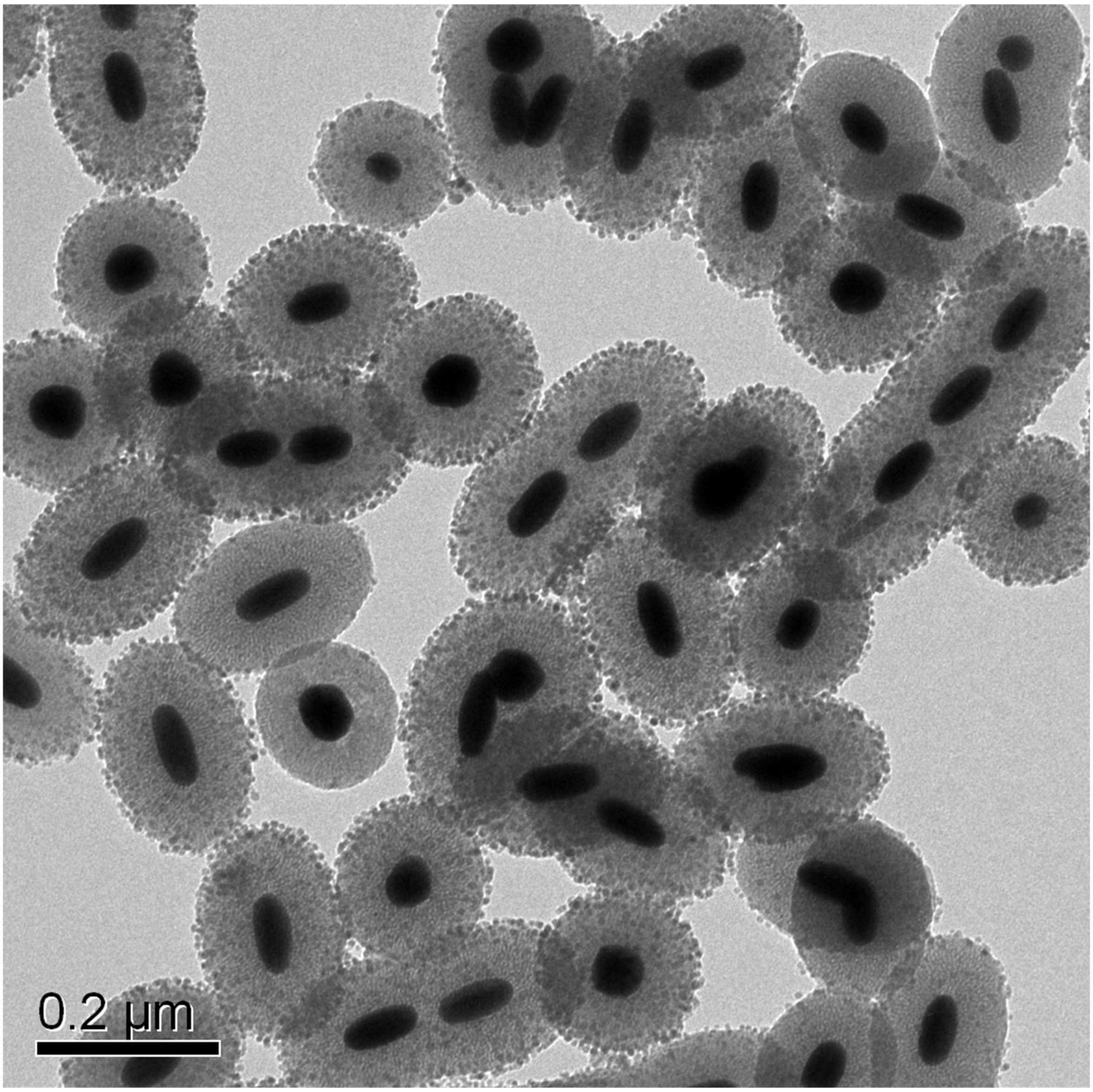


**Supplementary Figure 16** | Low magnification TEM image of IONP@AuBP nanohybrids photothermally synthesized at 200 °C for 180 min.

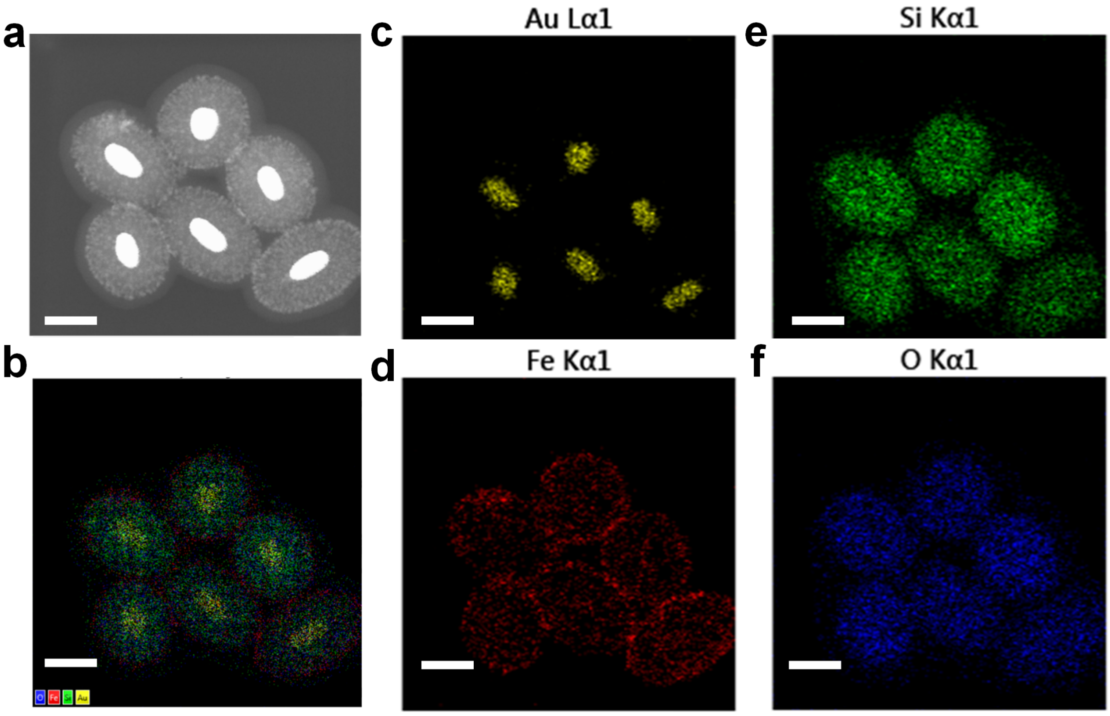


**Supplementary Figure 17 | EDS elemental mapping of IONP@AuBP nanohybrids under STEM mode. a,** STEM image clearly shows bright contrast of AuBPs as the core with small IONPs around as less bright dots, and **b,** overlapped image of individual elemental mapping of **c,** Au Lα1 (yellow), **d,** Fe Kα1 (red), **e,** Si Kα1 (green), **f,** O Kα1 (blue), over the same area showing the composite structure. Scale bars are 100 nm.

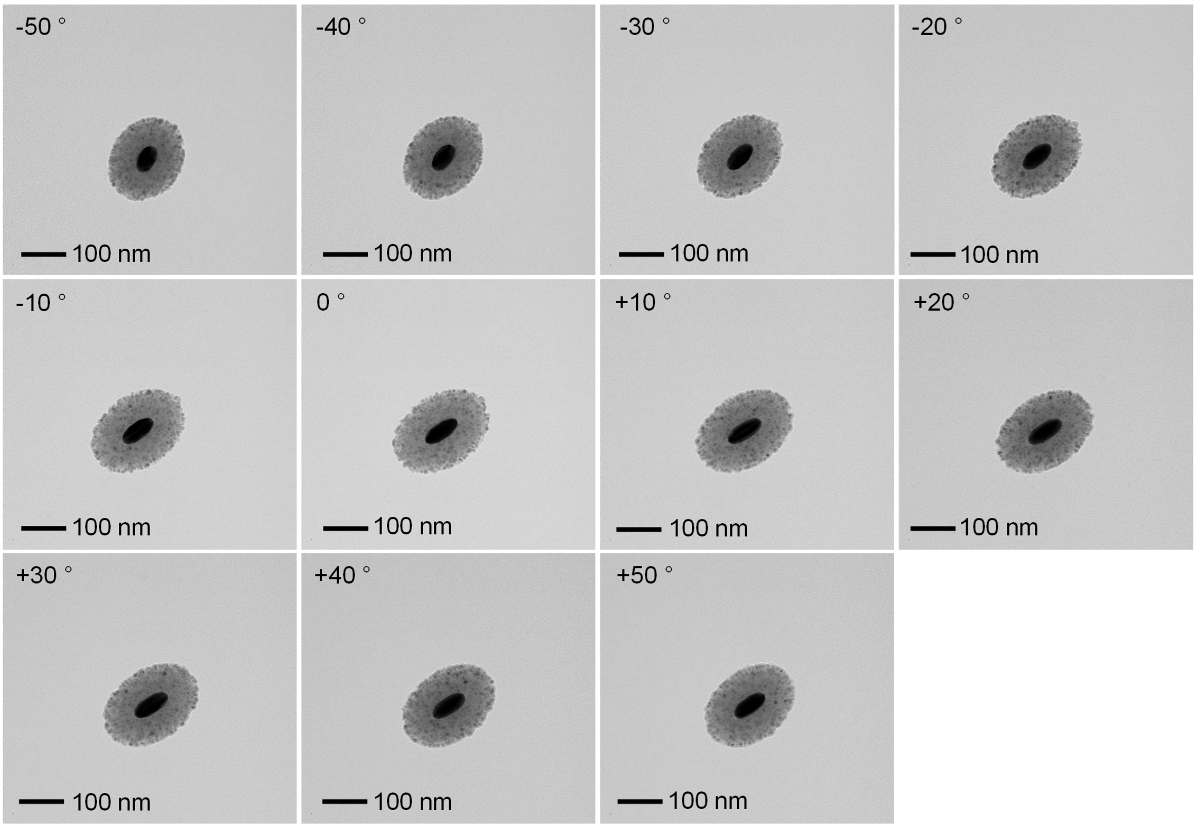


**Supplementary Figure 18** | TEM images of IONP@AuBP at different selected tilt angles (-50° to 50°) exhibiting the homogenous distribution of iron oxide nanoparticles over the AuBP.

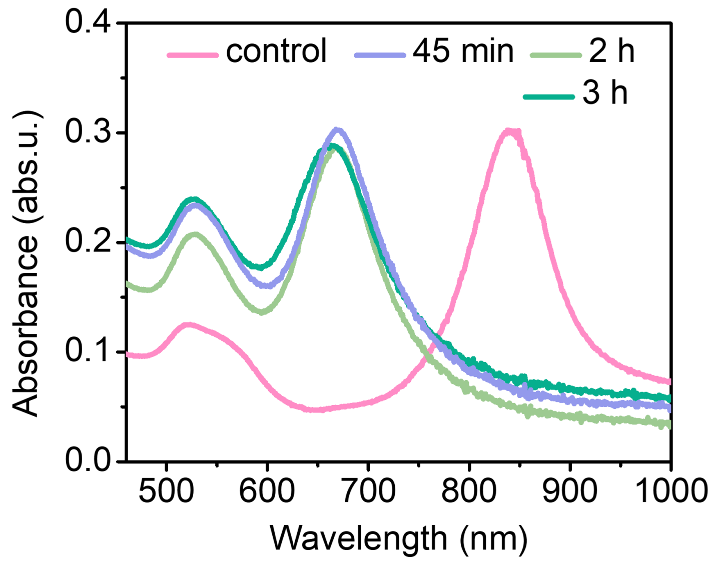


**Supplementary Figure 19** | UV-Vis absorbance spectra of the IONP@AuBP isolated at different time intervals during the photothermal synthesis at 200 °C (Supplementary Figure 14-16). The AuBP tips were degraded due to the photothermal heating with reduced LSPR ≈650 nm.  Control represents UV-Vis absorbance of the $SiO_2$@AuBP before the reaction. Source data are provided as a Source Data file.

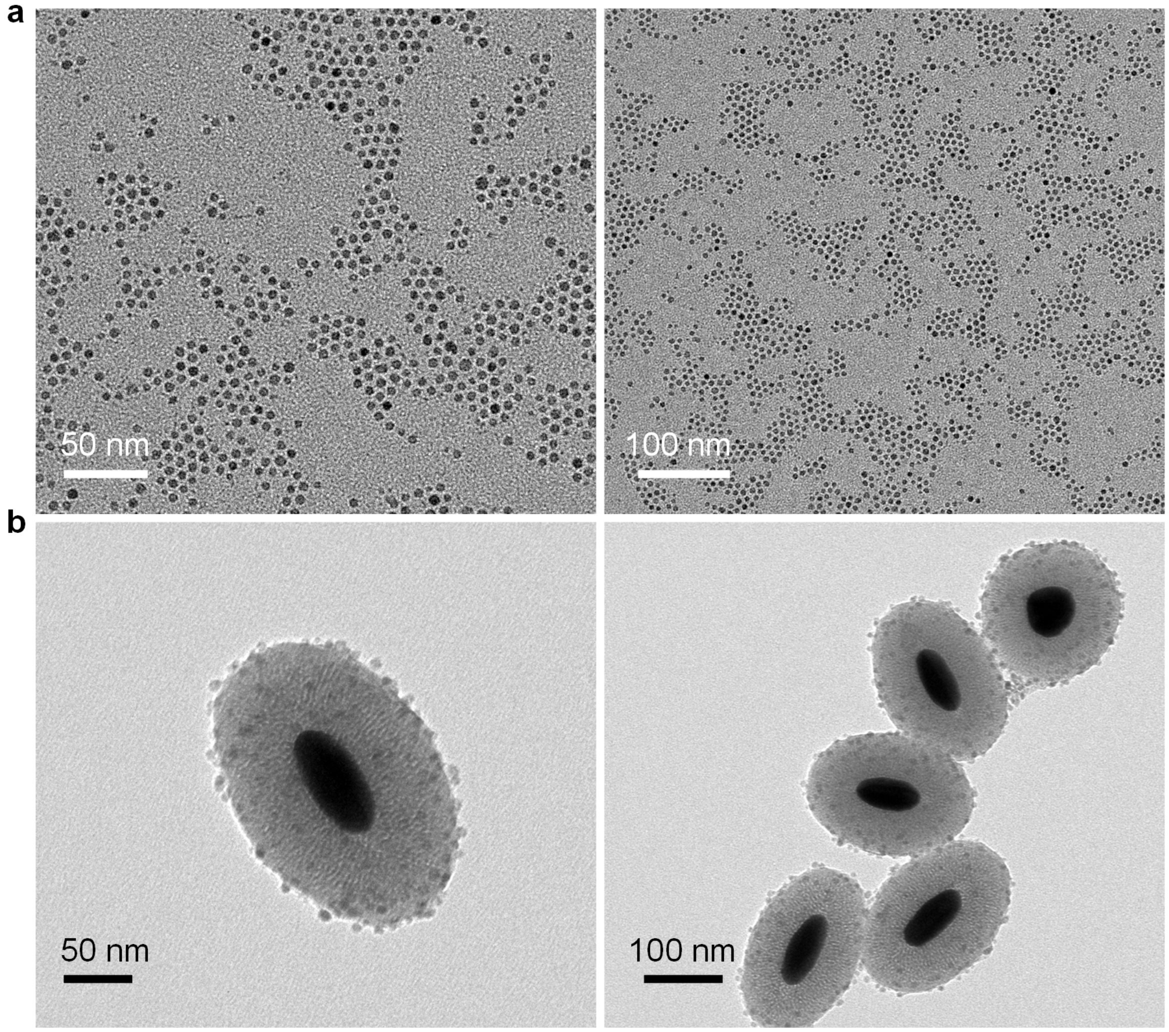


**Supplementary Figure 20** | TEM images of IONPs **a,** and corresponding IONP@AuBP nanohybrid **b,** photothermally synthesized at 180 °C for 2 h at different magnifications.

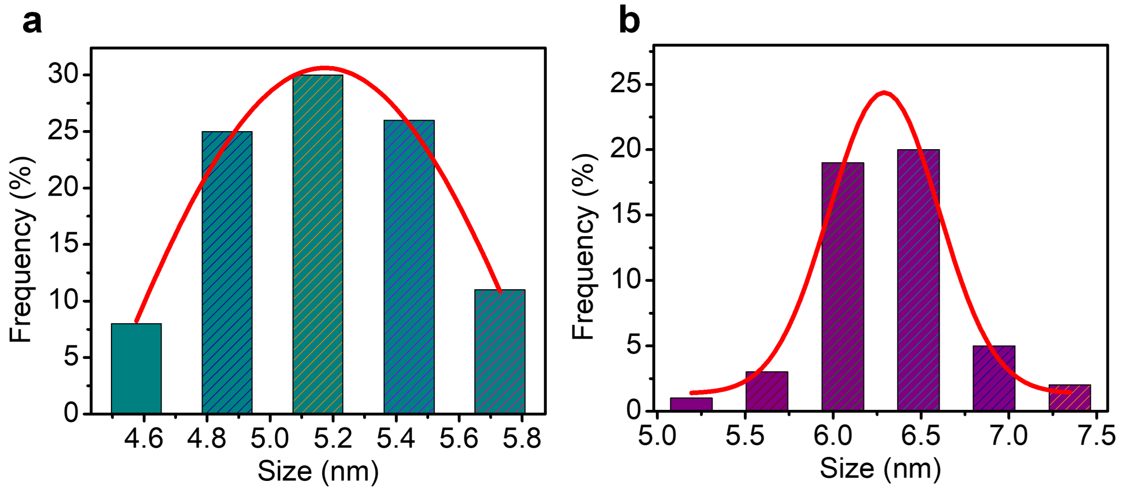


**Supplementary Figure 21** | **Size distribution of IONPs synthesized at photothermal temperatures of 180 °C for 2 h. a** (cyan)**,** IONPs, average size ≈5.2 nm, n=100, SD 0.3 nm, **b** (magenta)**,** IONPs on AuBPs, average size ≈6.3 nm, n=50, SD 0.4 nm. SD: standard

deviation. The distributions were obtained by analyzing 100 IONPs, and 50 IONPs on the AuBPs (n) chosen arbitrarily with ImageJ software, and fitted using gaussian function (red curve). Source data are provided as a Source Data file.

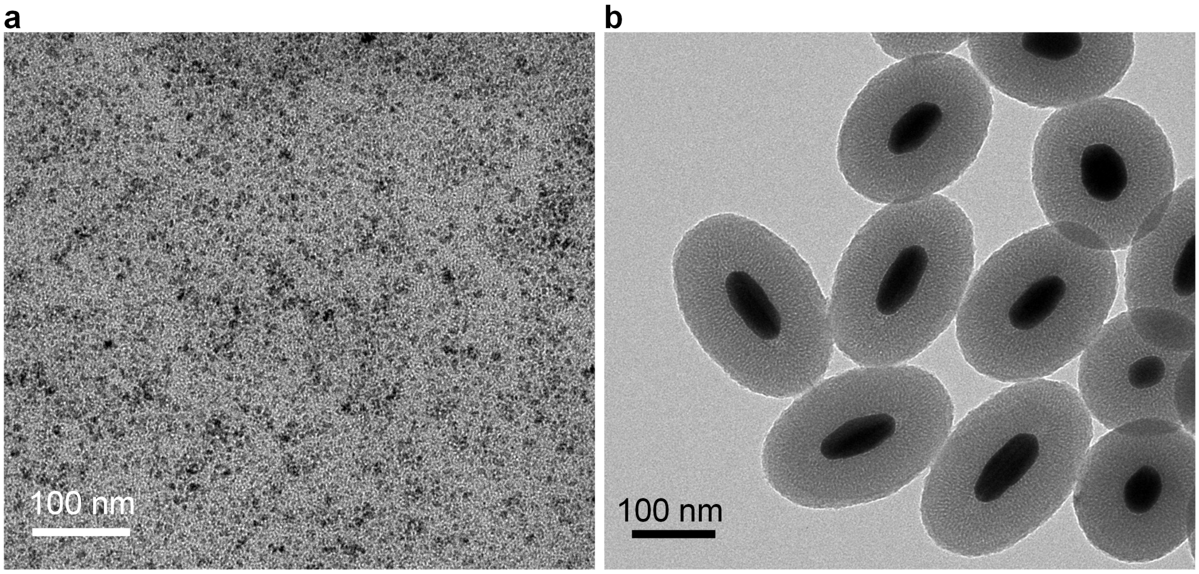


**Supplementary Figure 22** | TEM images of **a,** IONP, and **b,** AuBP, after conventional heating at 180 °C for 2 h in presence of AuBPs but without any light exposure. The nanoparticles obtained were smaller without well-defined morphological features and without any attachment on the AuBP surface leading to no nanohybrid formation as compared to the photothermally synthesized particles at the same conditions (Supplementary Figure 20).

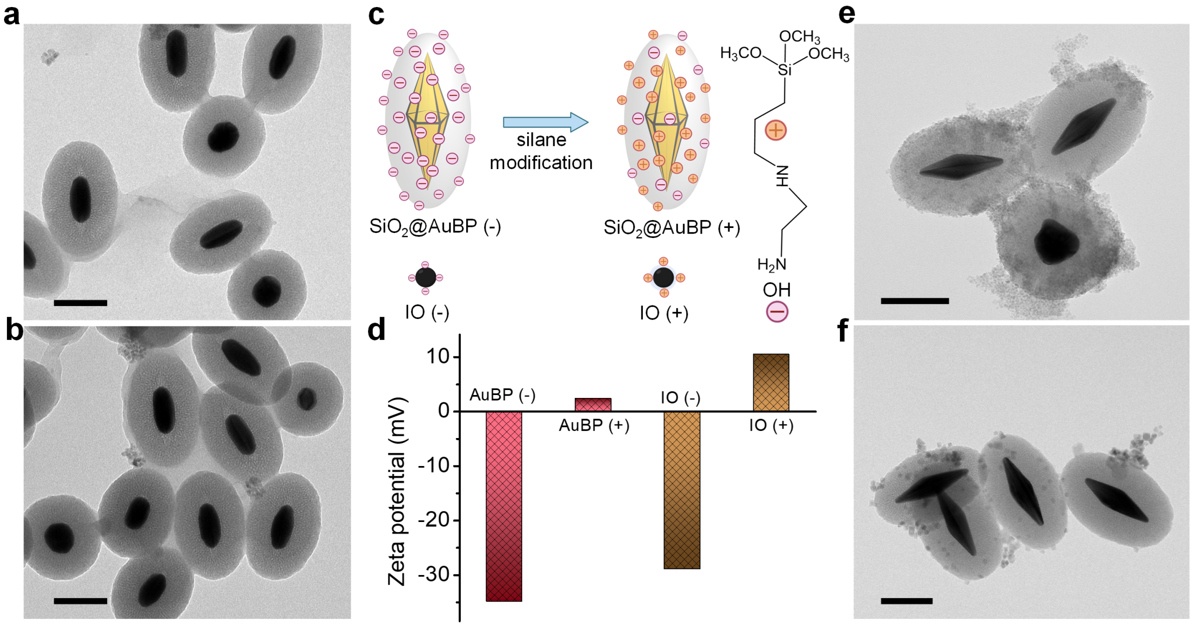


**Supplementary Figure 23** | TEM images of AuBPs after IONPs synthesis through conventional heating for 2 h in presence of AuBPs but without any light exposure at **a,** 210 °C, and **b,** 250 °C. Very few non-uniform aggregates of loosely bound IONPs were observed on the AuBP surface. **c,** Silane chemistry employed surface modification strategy of nanoparticles.

**d,** Change in zeta potential values (positive for AEAPS, negative for hydroxyl, darker color shades indicate higher values) post surface modification of the nanoparticles (reference 44). **e-f,** TEM images of oppositely charged nanoparticles in an attempt for synthesis of nanoparticle-$SiO_2$@AuBP hybrids, **e,** through AuBP(+)/IO(-) and **f,** through AuBP(-)/IO(+). Scale bars are 100 nm. Source data are provided as a Source Data file.

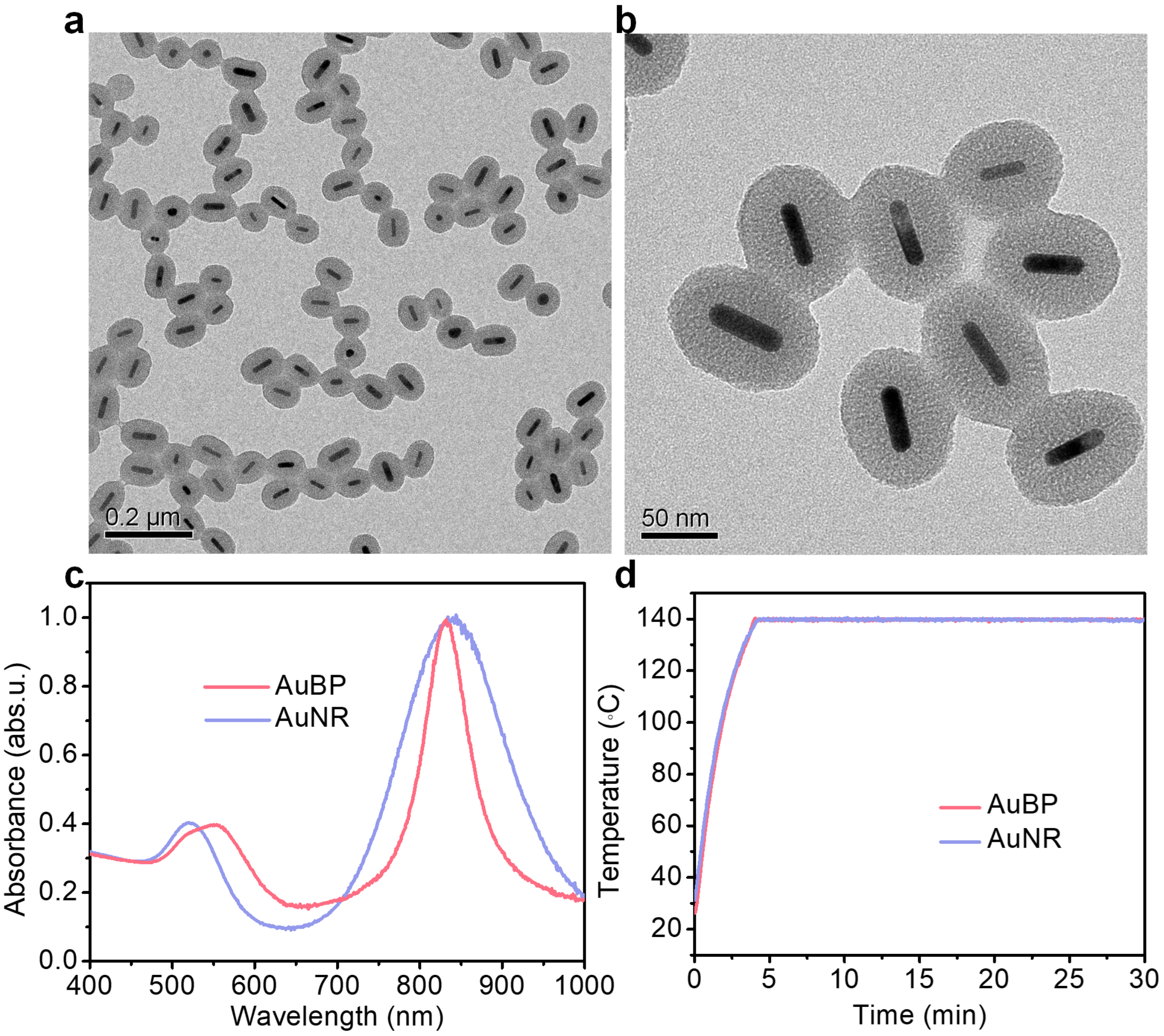


**Supplementary Figure 24 | a-b,** TEM images of silica-coated gold nanorods ($SiO_2$@AuNR) at **a,** low, and **b,** high magnifications. **c,** Normalized UV-Vis absorption spectra of silica-coated AuBP and AuNR in ethanol, and **d,** corresponding temperature profile under 850 nm light irradiation in DMF (4 OD measured at 850 nm). Source data are provided as a Source Data file.

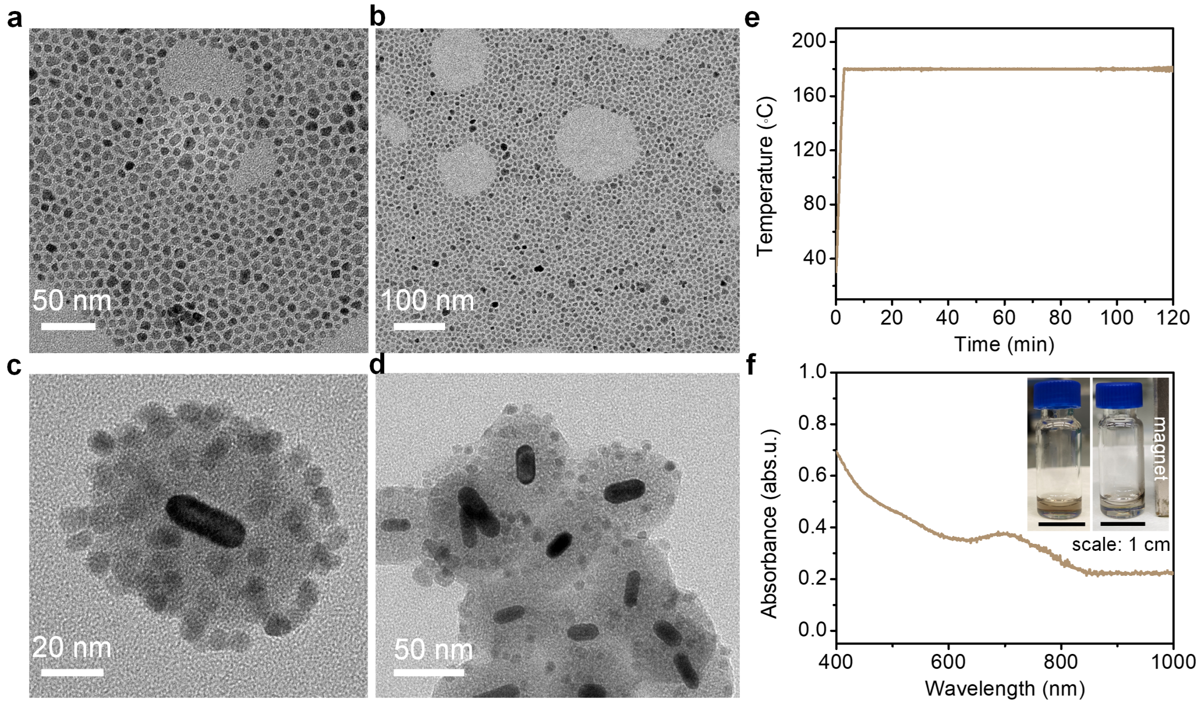


**Supplementary Figure 25 | a-b,** TEM images of iron oxide nanoparticles (IONPs, ≈4-6 nm), and **c-d,** corresponding IONP@AuNR$_{850}$ (IONPs, ≈5-7 nm) at different magnifications, synthesized with photothermal heating at 180 °C for 2 h under 850 nm light irradiation. **e,** Temperature profile of the reaction, and **f,** UV-Vis absorption spectrum of the IONP@AuNR with magnetic response (inset). Source data are provided as a Source Data file.

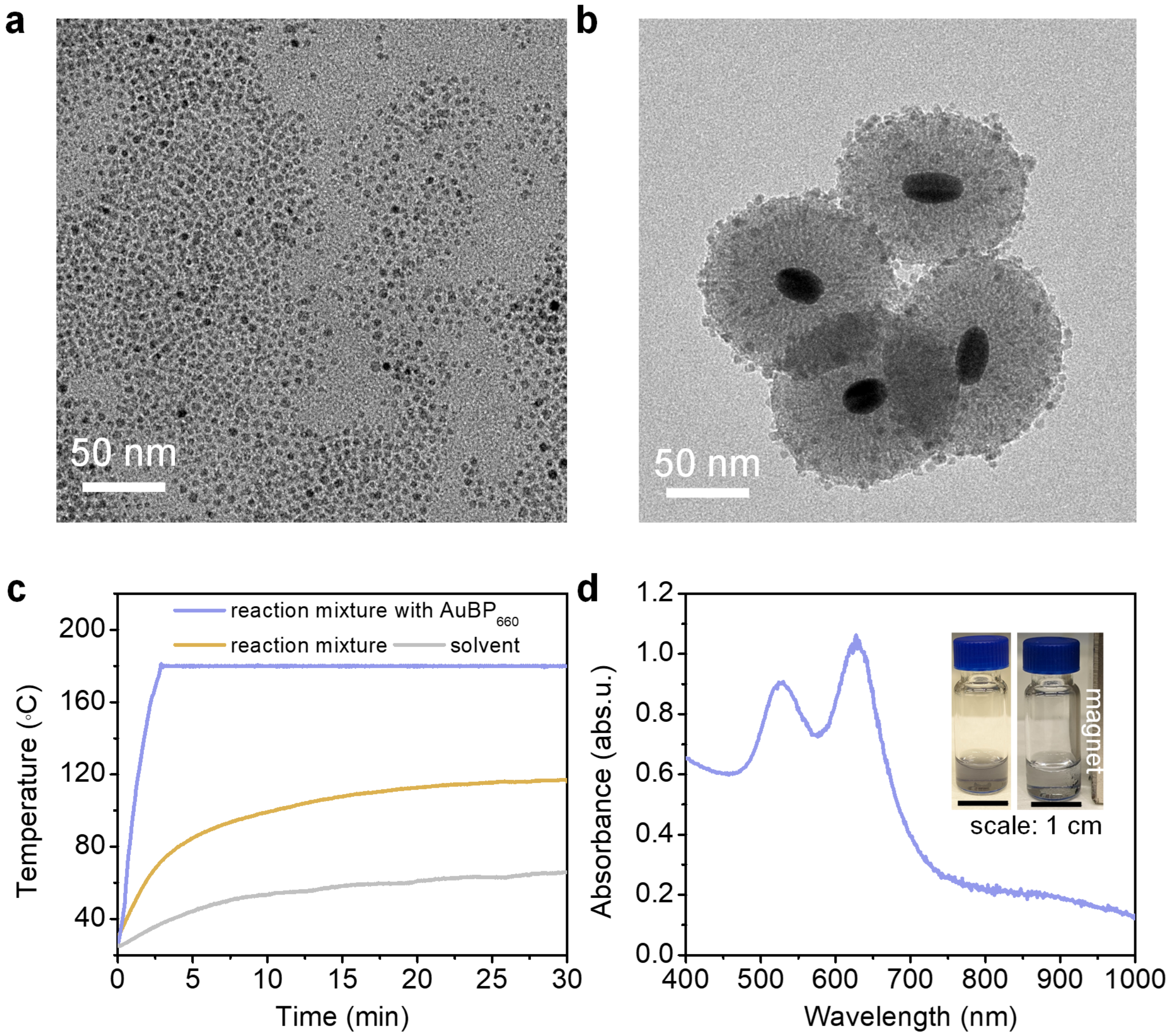


**Supplementary Figure 26 | a-b,** TEM images of **a,** iron oxide nanoparticles (IONPs, ≈4-5 nm) and **b,** corresponding IONP@AuBP$_{660}$ (IONPs, ≈5-6 nm), synthesized with photothermal heating at 180 °C for 2 h under 660 nm light irradiation. **c,** Temperature profile for the control reactions under the same photothermal conditions highlighting the leading role of the AuBPs. **d,** UV-Vis absorption spectrum of the IONP@AuBP$_{660}$ with magnetic response (inset). Source data are provided as a Source Data file.

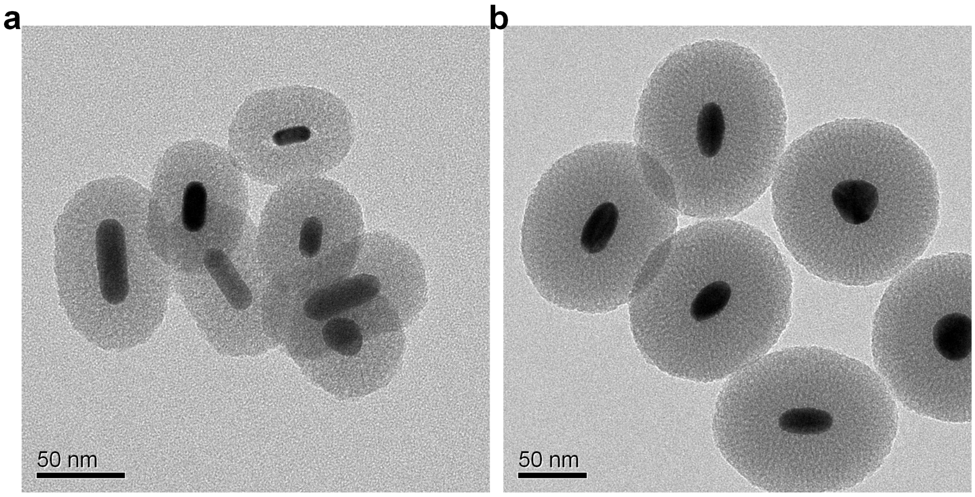


**Supplementary Figure 27** | TEM images of **a,** $AuNR_{850}$ and **b,** $AuBP_{660}$ post photothermal reaction at 180 °C for 4 h.

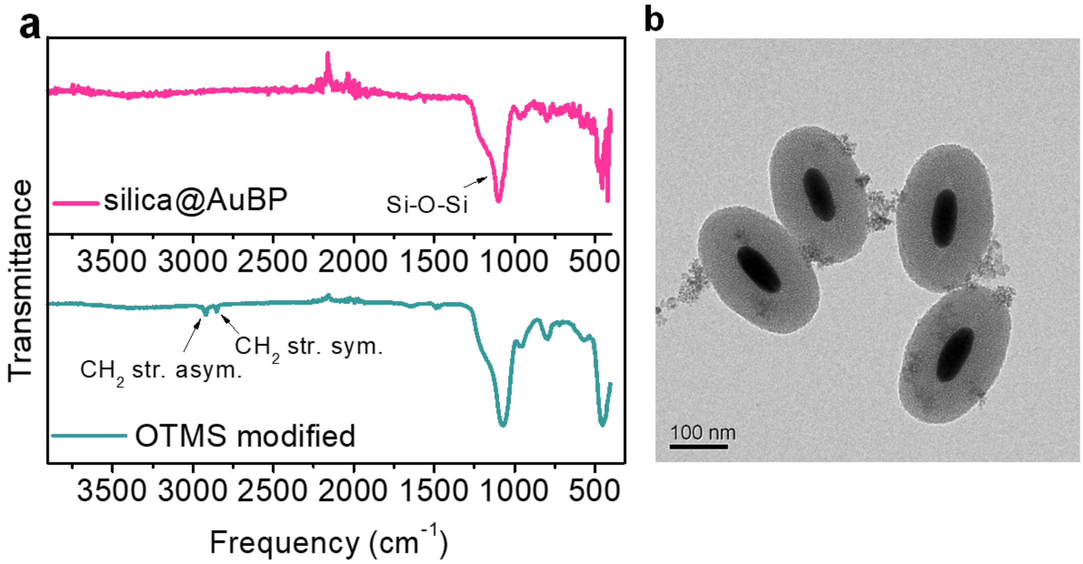


**Supplementary Figure 28** | **FTIR characterization of octadecyltrimethoxysilane (OTMS) modified AuBP. a,** The FTIR spectra show appearance of characteristics peaks of $CH_2$ asymmetric and symmetric stretching (arrow) due to long alkyl chain of the OTMS on the AuBP surface. **b,** Additional TEM image of OTMS-modified AuBPs after photothermal synthesis of IONPs at 200 °C for 2 h, exhibiting no nanohybrid formation. Very few non-uniform aggregates of loosely bound IONPs were observed on the AuBP surface. Source data are provided as a Source Data file.

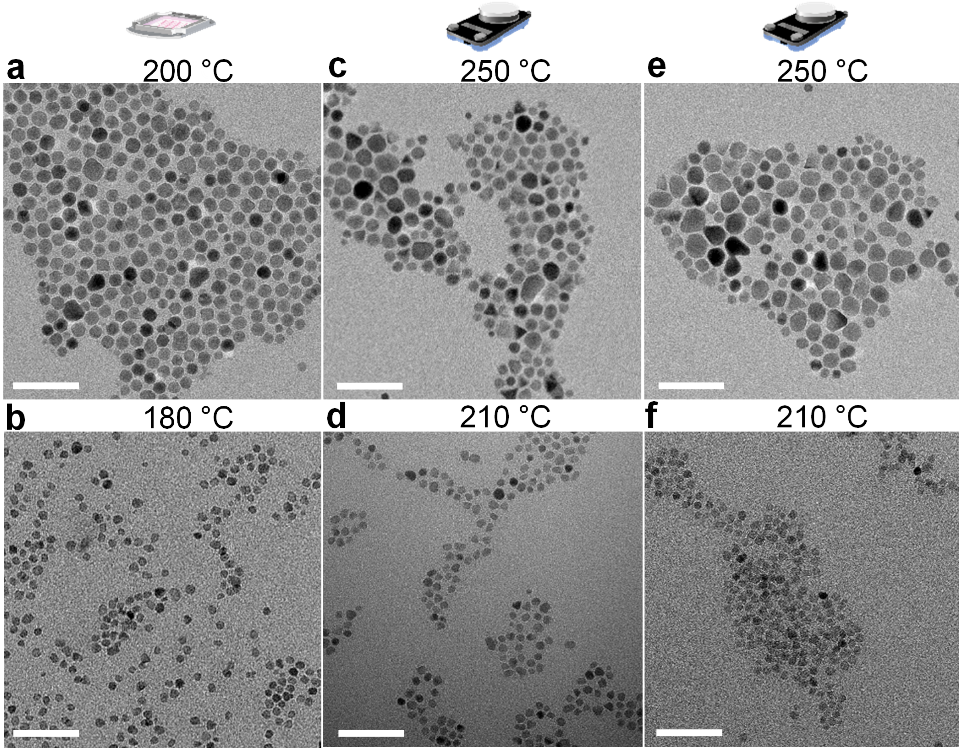


**Supplementary Figure 29 | Comparison of the size of the nanoparticles formed under photothermal conditions as described in Supplementary Figure 3, and under the same but conventional heating conditions.** TEM images of IONPs formed (**a-b**) under photothermal conditions, (**c-d**) under conventional heating conditions, and (**e-f**) under conventional heating condition with **e,** initial temperature ramp ≈22 °C/min and **f,** ≈36 °C/min. Photothermal conditions demonstrate higher growth of the nanoparticles with a temperature difference of 30-50 °C. Scale bars are 50 nm.

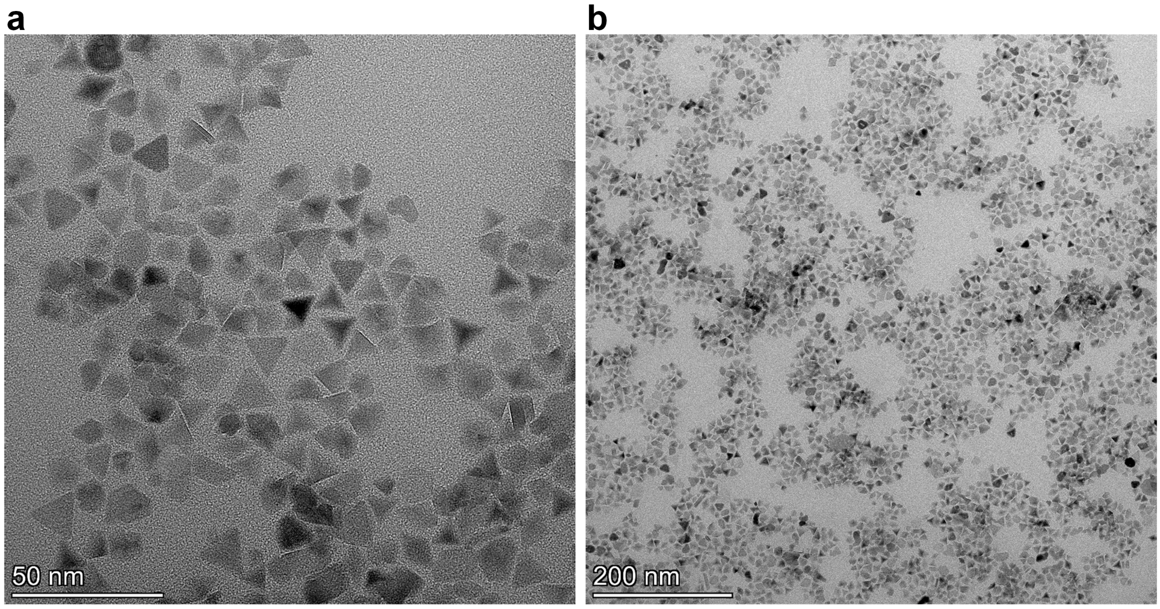


**Supplementary Figure 30** | Additional TEM images of photothermally synthesized triangular iron oxide nanoparticles (TIONPs) at **a,** high, and **b,** low magnifications.

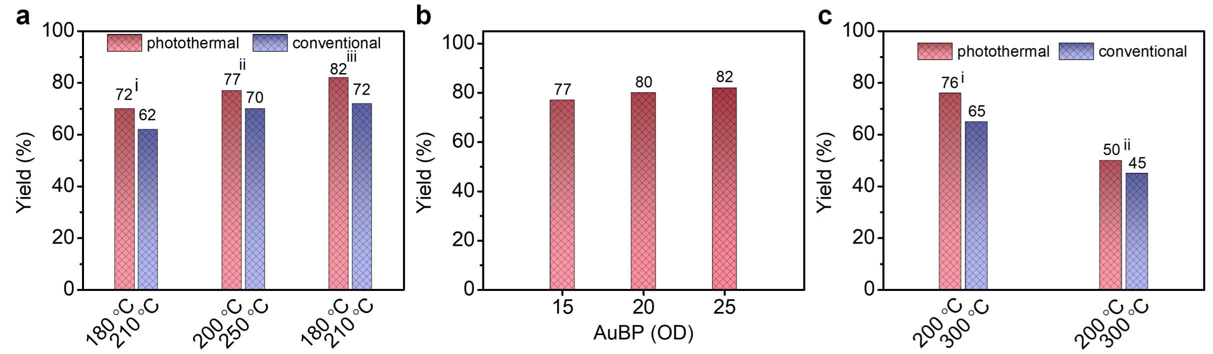


**Supplementary Figure 31 | Synthetic yield of nanoparticles under photothermal (with 15 OD AuBPs) and conventional reactions. a,** Iron oxide nanoparticle synthesis for **i,** ≈4-6 nm particles, **ii,** ≈10 nm particles, **iii,** ≈6-8 nm triangular particles between 180-250 °C. **b,** Iron oxide nanoparticle synthesis at a photothermal temperature of 200 °C with different concentrations (15-25 OD measured at 850 nm) of AuBPs with a slightly greater yield at higher AuBP concentrations. **c,** Iron oxide nanoparticle synthesis corresponding to **i,** ≈2-4 nm particles, and **ii,** ≈15 nm nanoplates at 200-300 °C. Source data are provided as a Source Data file.

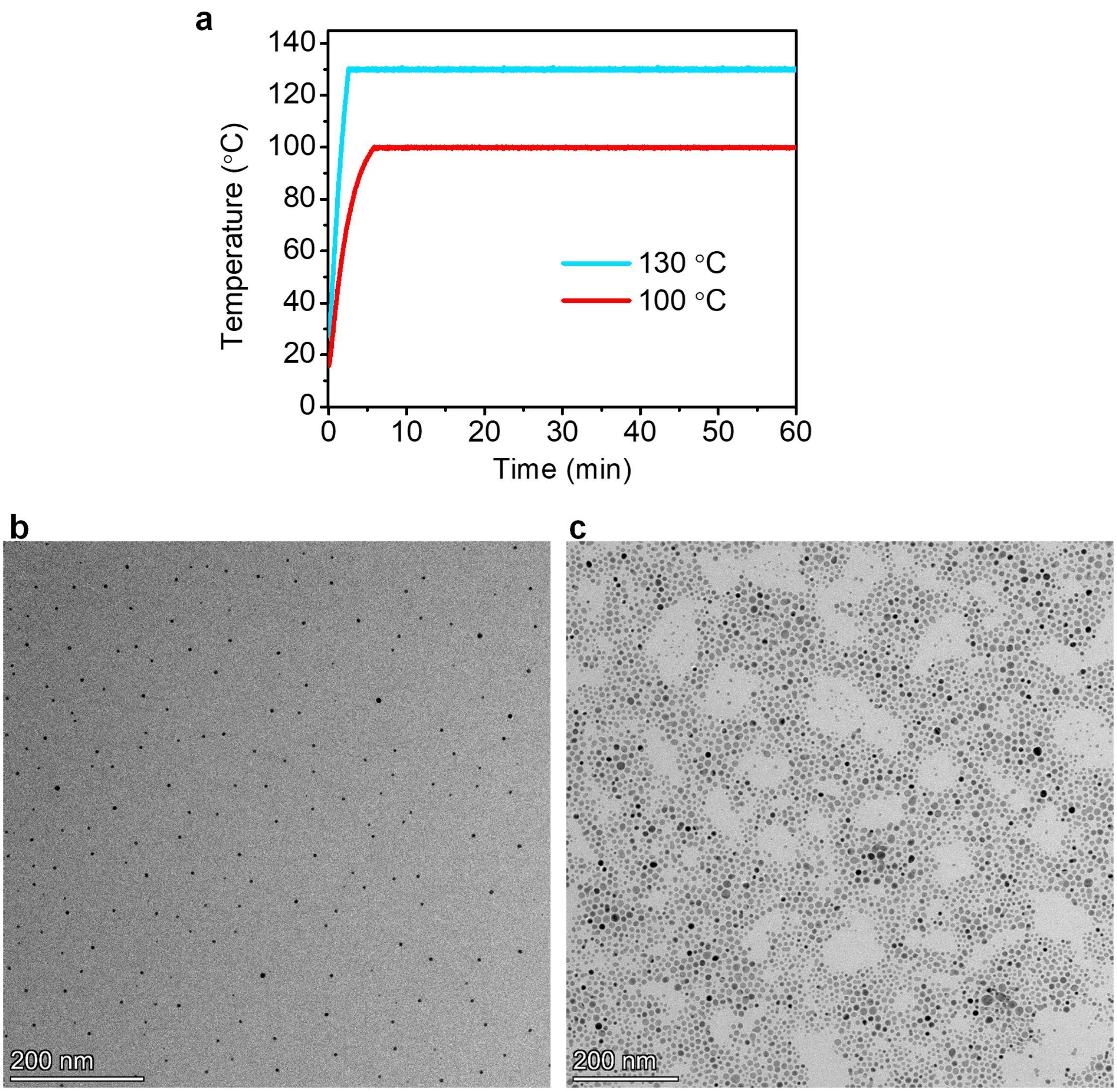


**Supplementary Figure 32 | a,** Temperature profile, and **b-c,** Low magnification TEM images of the silver nanoparticles synthesized in oleylamine at two different photothermal temperatures, **b,** 100 °C, and **c,** 130 °C. Source data are provided as a Source Data file.

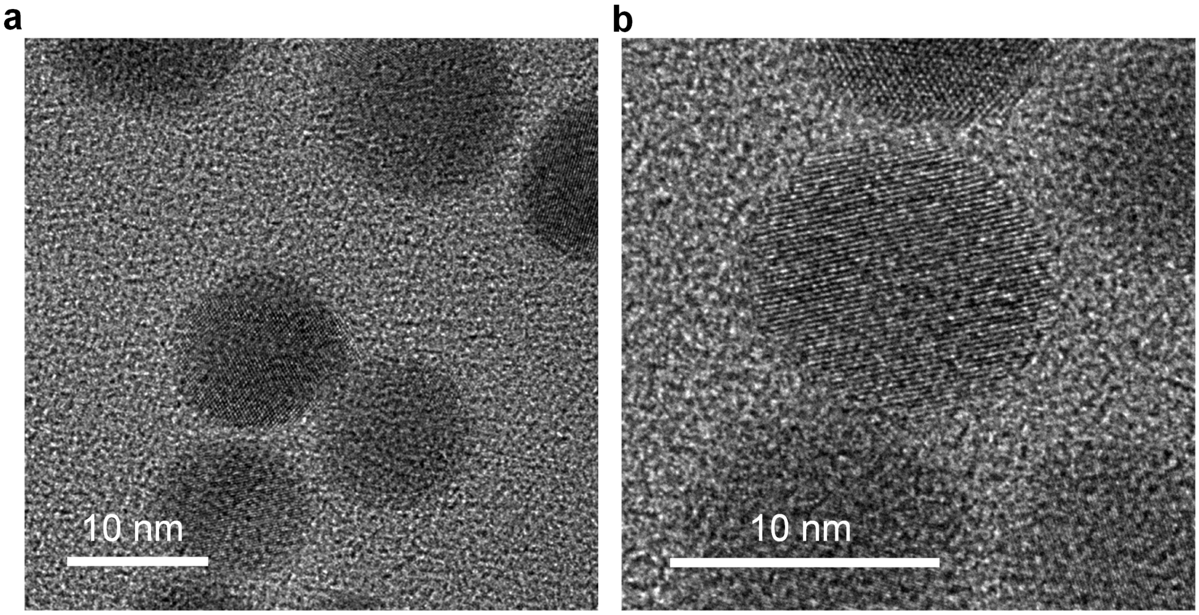


**Supplementary Figure 33** | **a-b,** High-resolution TEM (HR-TEM) images of AgNPs at different magnifications.

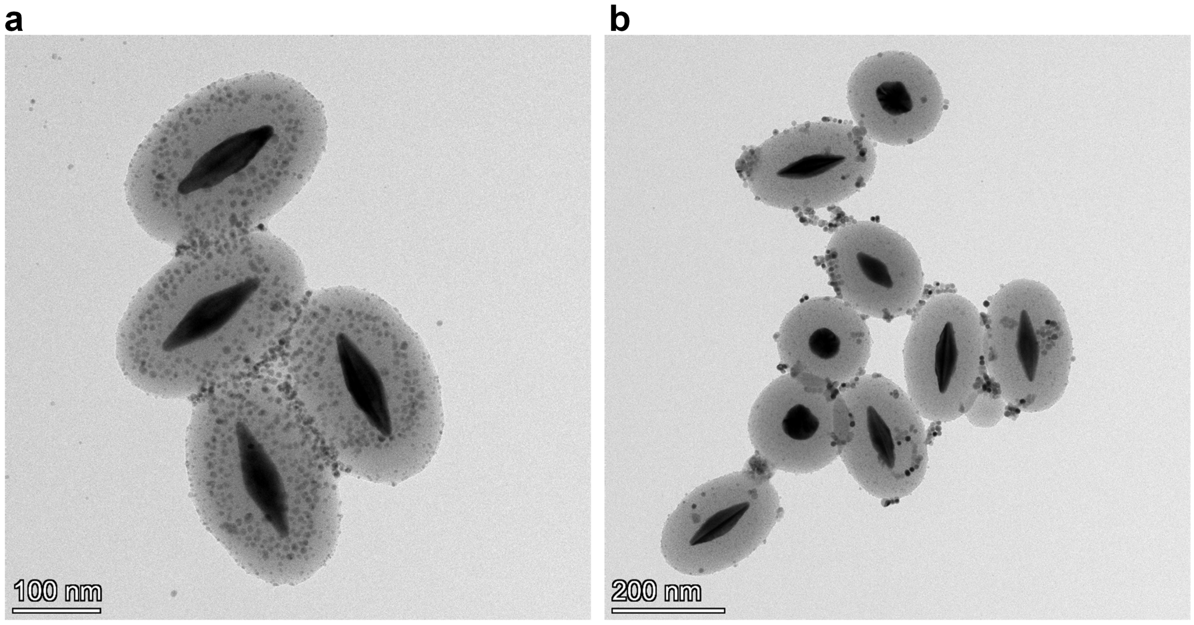


**Supplementary Figure 34** | Low magnification TEM images of the silver nanoparticles decorated AuBPs after the photothermal synthesis at **a,** 100 °C, and **b,** 130 °C.

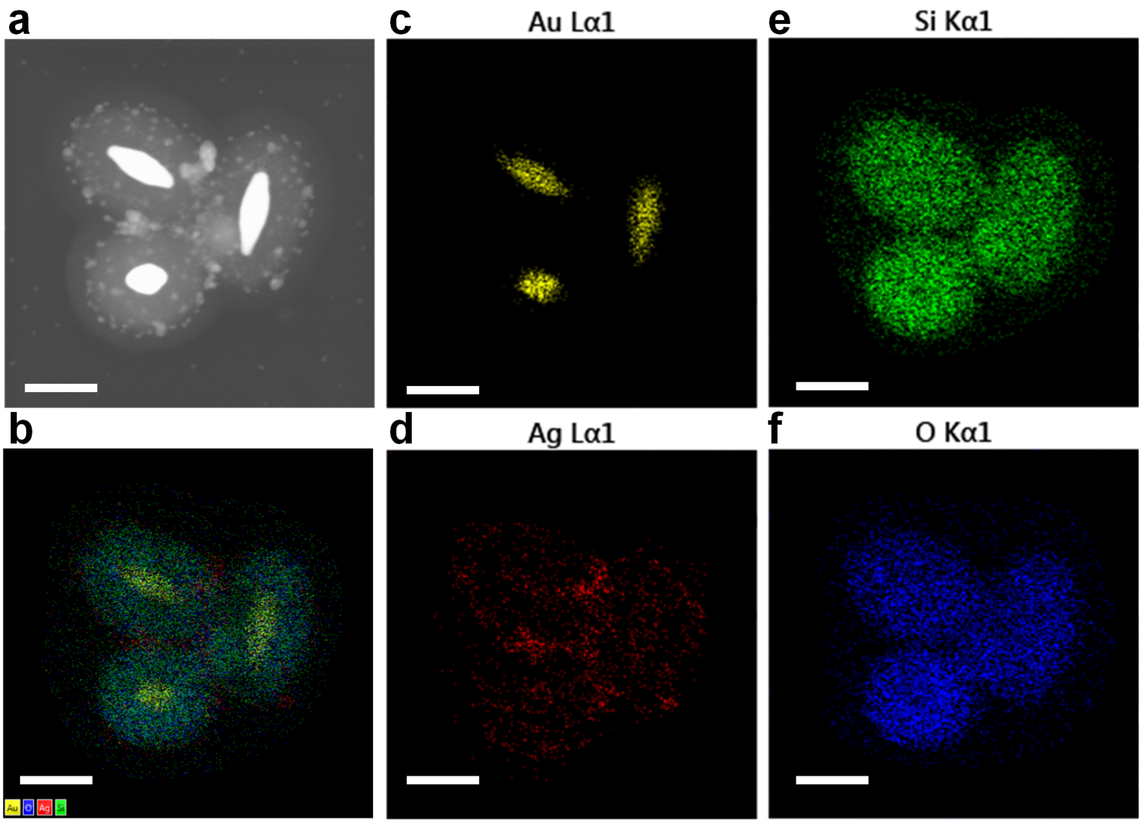


**Supplementary Figure 35 | EDS elemental mapping of AgNPs decorated AuBPs under STEM mode. a,** STEM image clearly shows bright contrast of AuBPs as the core with small AgNPs around as discreate bright dots, and **b,** overlapped image of the individual elemental mapping of **c,** Au Lα1 (yellow), **d,** Ag Lα1 (red), **e,** Si Kα1 (green), **f,** O Kα1 (blue), over the same area showing the composite structure. Scale bars are 100 nm.

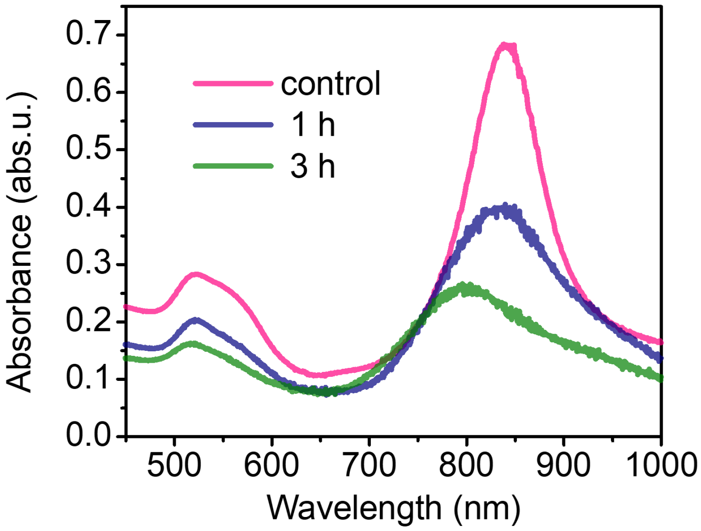


**Supplementary Figure 36** | UV-Vis absorption spectra of AgNPs decorated AuBPs obtained at a photothermal temperature of 130 °C for 1-3 h. Control represents UV-Vis absorbance of the $SiO_2$@AuBP before the reaction. Source data are provided as a Source Data file.

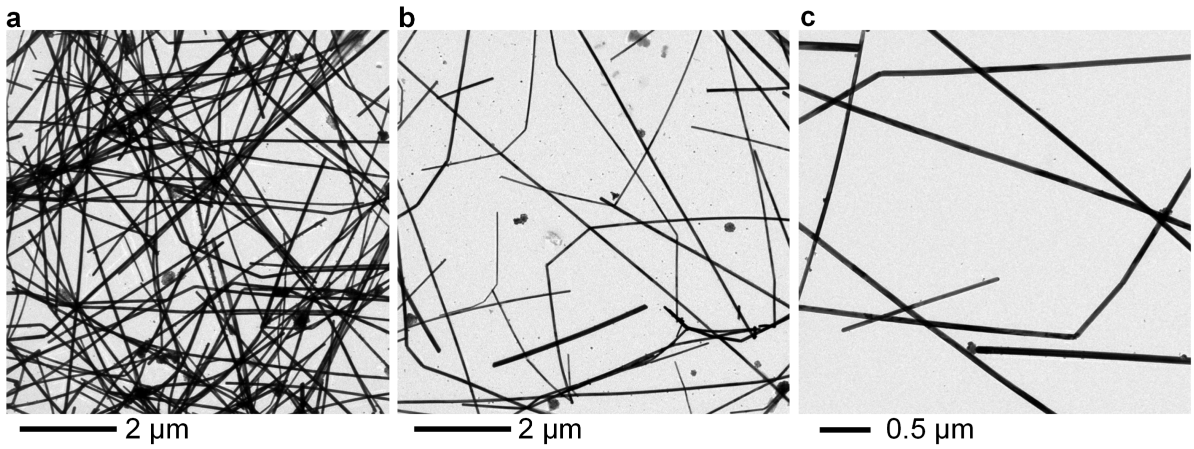


**Supplementary Figure 37 | a-c,** TEM images of AgNWs at different magnifications synthesized via conventional heating in ethylene glycol at 140 °C.

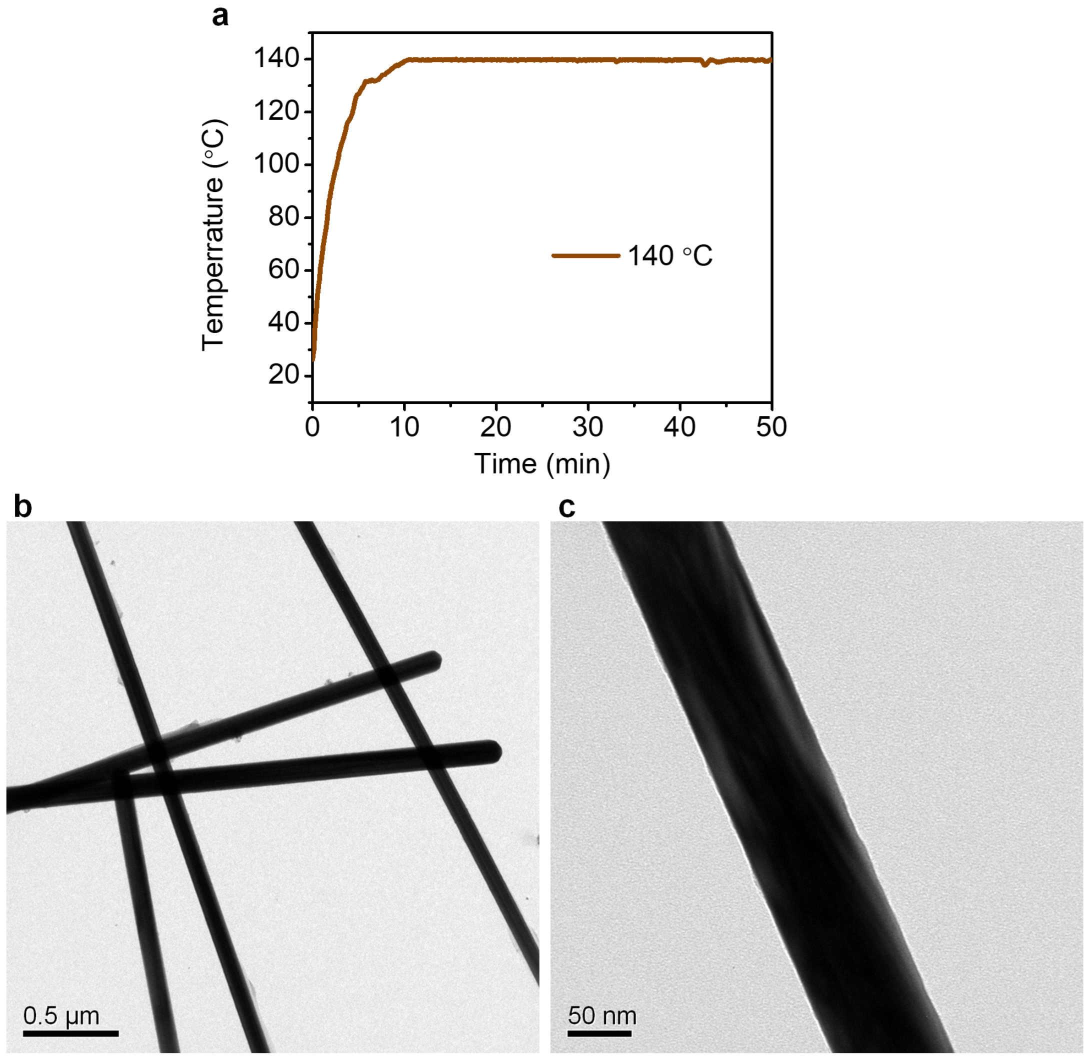


**Supplementary Figure 38 | a,** Temperature profile, and **b-c,** TEM images of the silver nanowires at two different magnifications synthesized via photothermal heat in ethylene glycol. Source data are provided as a Source Data file.

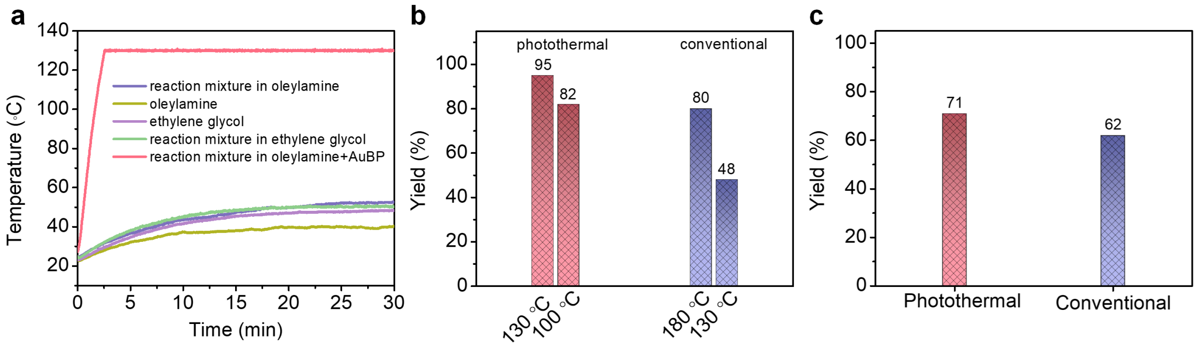


**Supplementary Figure 39** | **a,** Temperature profile for control reactions only without gold bipyramids in different solvents exhibiting insignificant contribution of the reaction mixture/solvents towards photothermal synthesis of silver nanoparticle under 850 nm light irradiation. Notably, no nanoparticle was isolated in the control experiments without AuBPs. **b-c,** Silver nanoparticle **b,** and silver nanowire **c,** synthesized in different reaction conditions exhibiting consistent and greater yield under photothermal conditions (with 15 OD AuBPs measured at 850 nm) at lower temperatures. Source data are provided as a Source Data file.

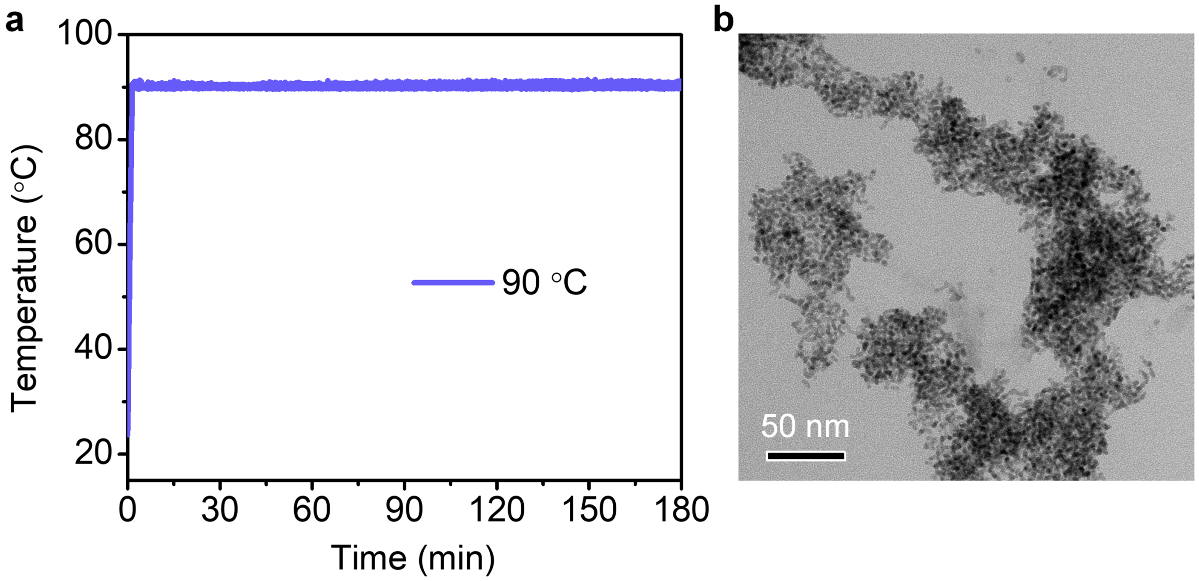


**Supplementary Figure 40** | **a,** Temperature profile, and **b,** additional TEM image of elongated clustered palladium nanoparticles synthesized at a photothermal temperature of 90 °C in toluene. Source data are provided as a Source Data file.

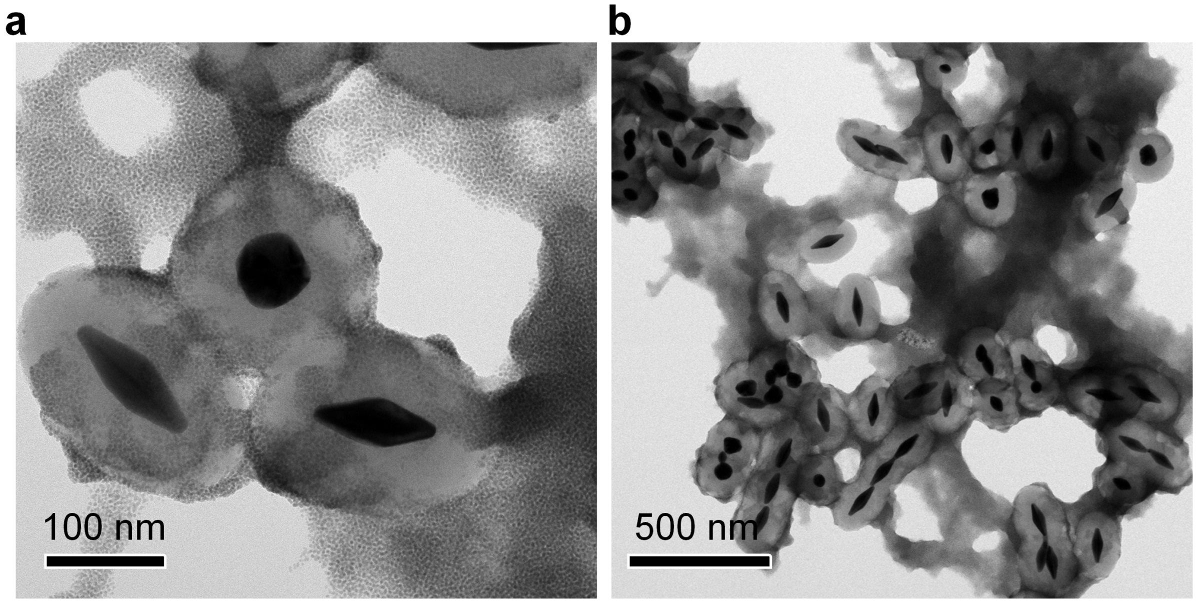


**Supplementary Figure 41** | Additional TEM images at **a,** high, and **b,** low magnifications showing elongated clustered palladium nanoparticles around the $SiO_2$@AuBPs synthesized at a photothermal temperature of 90 °C in toluene.

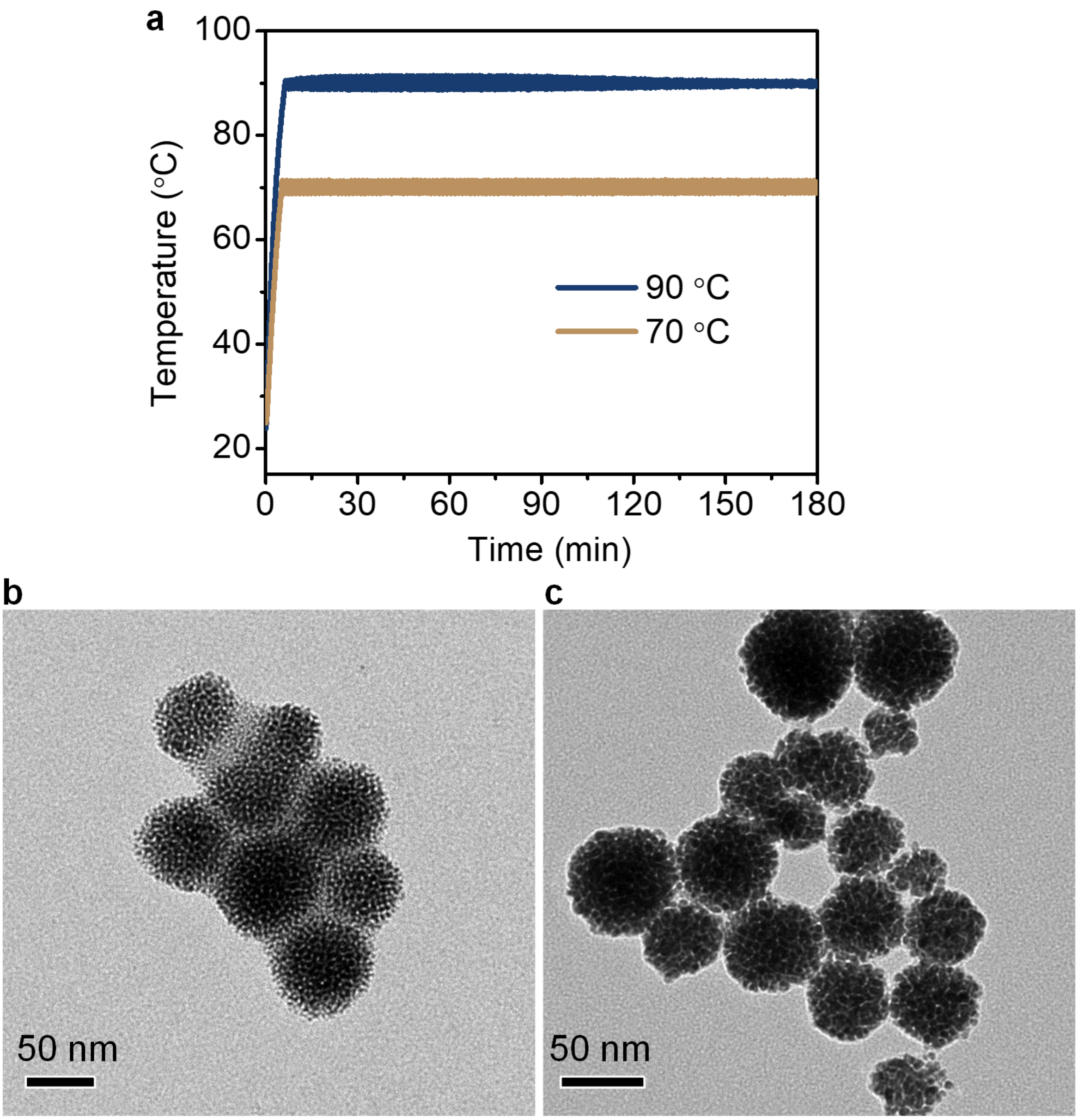


**Supplementary Figure 42 | a,** Temperature profile for photothermal synthesis of Pd-nanoassembly in ethanol. Additional TEM images of the Pd-nanoassembly synthesized photothermally in ethanol at **b,** 70 °C, and **c,** 90 °C. Source data are provided as a Source Data file.

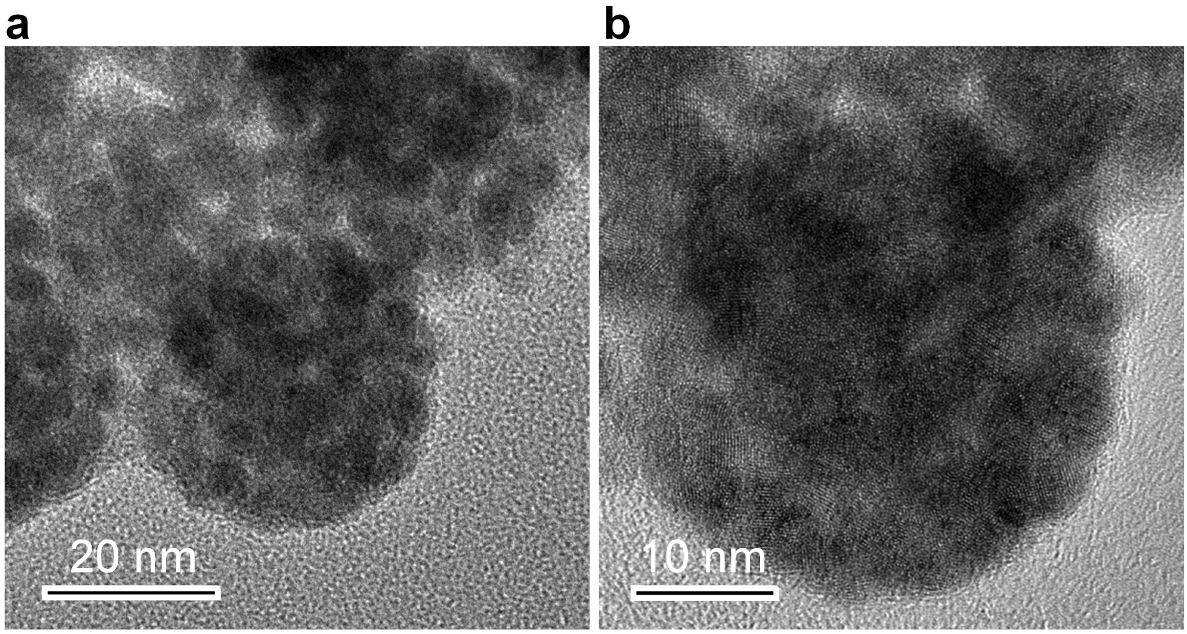


**Supplementary Figure 43** | **a-b,** High-resolution TEM (HR-TEM) images of Pd-nanoassemblies at different magnifications showing individual crystalline lattice fringes of small PdNPs inside the assembly.

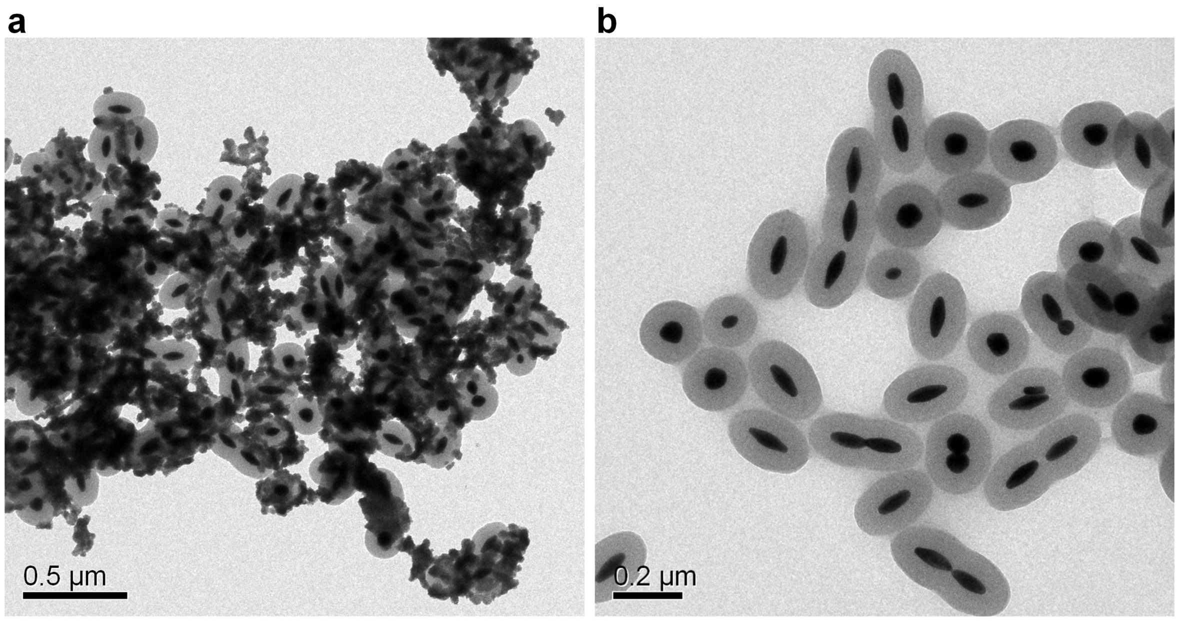


**Supplementary Figure 44** | Low magnification TEM images of AuBPs exhibiting **a,** Pd-assembly on the AuBPs that were obtained through photothermal heating in ethanol at 70 °C, while **b,** no assemblies were seen on the AuBP surface at 90 °C.

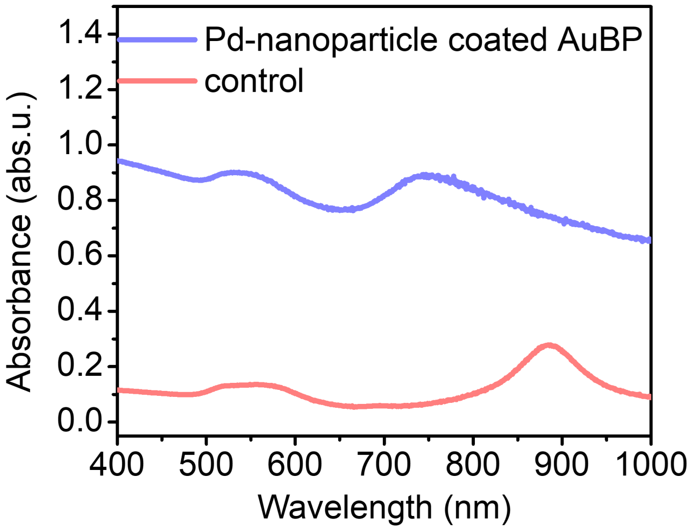


**Supplementary Figure 45** | UV-Vis absorption spectra of Pd-assembly covered AuBPs obtained at photothermal temperature of 70 °C in ethanol. Source data are provided as a Source Data file.

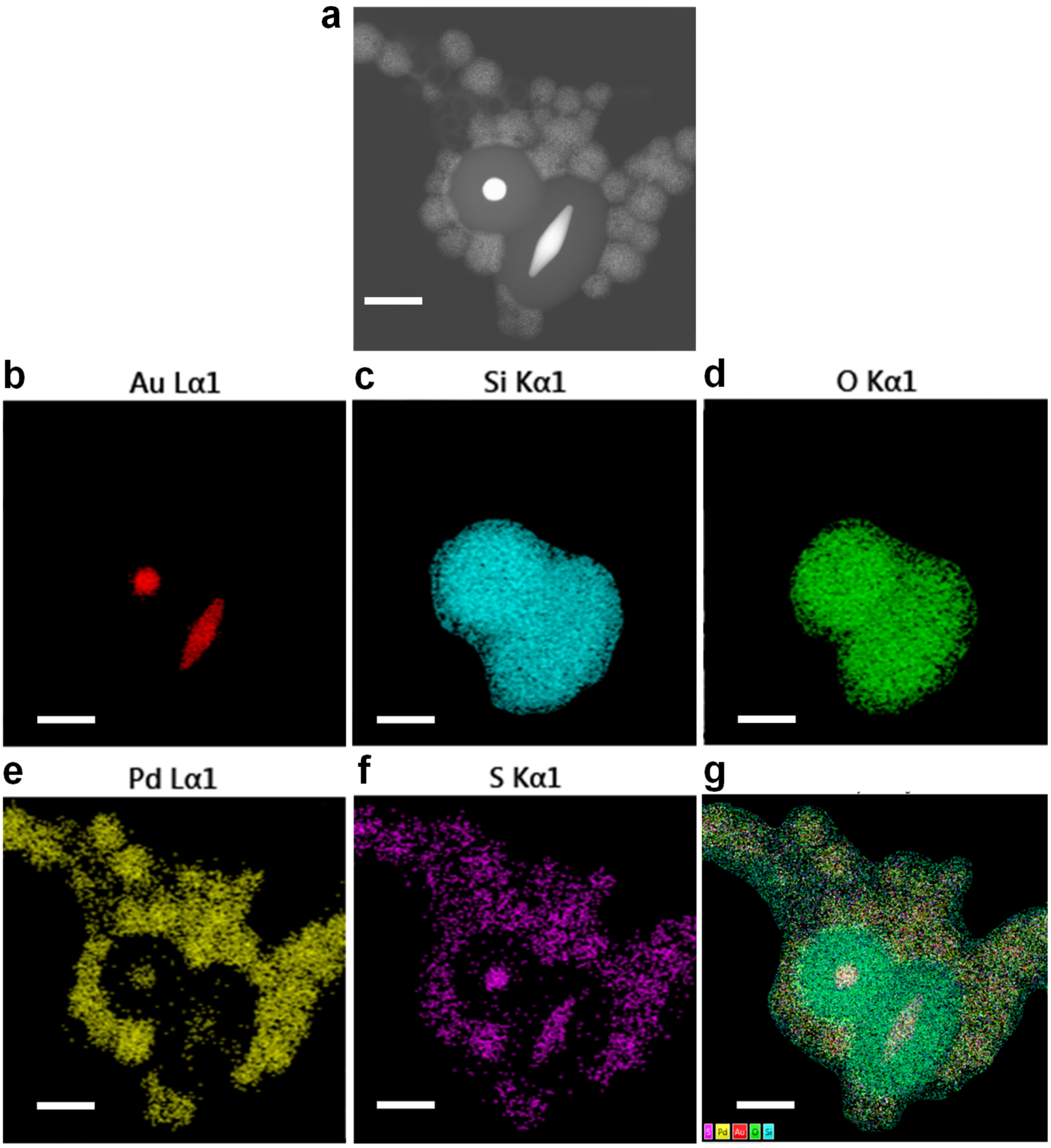


**Supplementary Figure 46 | EDS elemental mapping of Pd-nanoassembly on the AuBP under STEM mode. a,** STEM image clearly shows bright contrast of AuBPs as the core with assemblies of palladium around. Individual elemental mapping of **b,** Au Lα1 (red), **c,** Si Kα1 (blue), **d,** O Kα1 (green), **e,** Pd Lα1 (yellow), and **f,** S Kα1 (pink) over the same area with **g,** overlapping of STEM, and all of the individual mappings exhibits the supported composite structure. Scale bars are 100 nm.

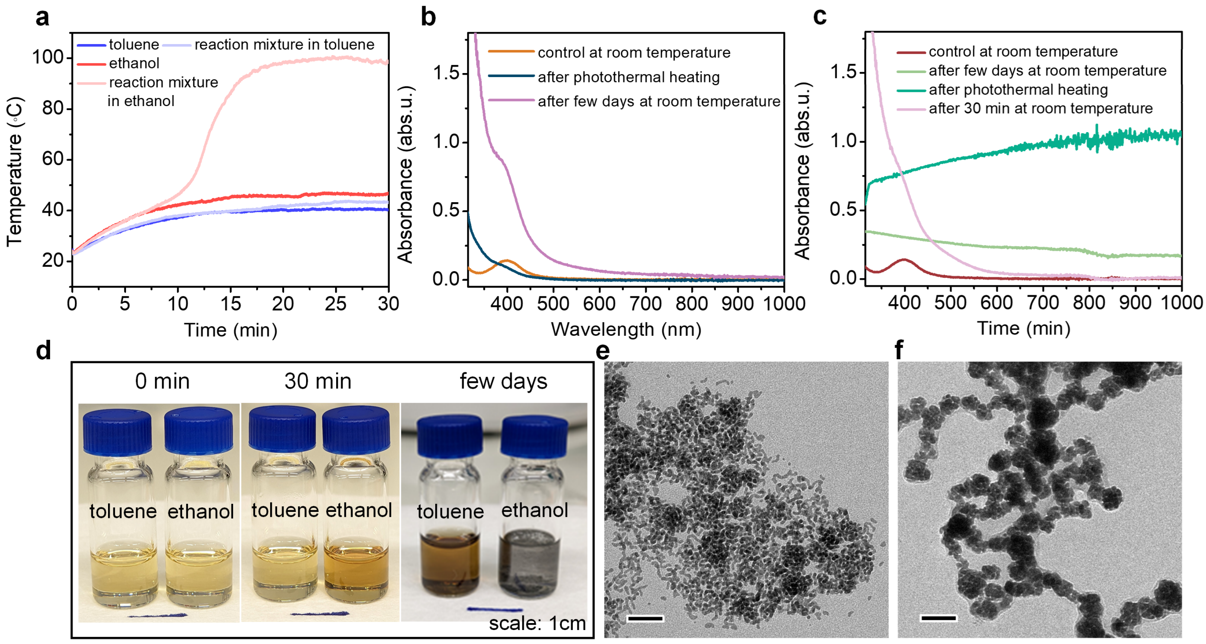


**Supplementary Figure 47** | **a-c,** Temperature profile **a,** and UV-Vis absorption spectra **b-c,** (in toluene **b,** in ethanol **c,**) for control reactions only without gold bipyramids in different solvents for photothermal synthesis of palladium nanoparticle under 850 nm light irradiation. In toluene, insignificant contribution of the reaction mixture/solvents towards photothermal synthesis of palladium nanoparticles, and notably, no nanoparticle was isolated without AuBPs. **d,** Digital images showing stability of the palladium precursor in different solvents for different time intervals. **e-f,** TEM images of palladium nanoparticle isolated from the reaction in ethanol which was kept at room temperature for a week **e,** and synthesized under photothermal condition without AuBPs under light **f**. Scale bars are 50 nm. Source data are provided as a Source Data file.

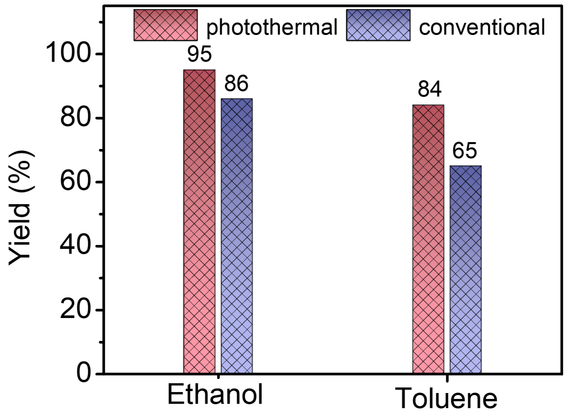


**Supplementary Figure 48** | Palladium nanoparticle synthesis in different reaction conditions exhibiting slightly higher nanoparticle yield in the ethanolic medium and under photothermal conditions (with 2 OD AuBPs measured at 850 nm). Source data are provided as a Source Data file.

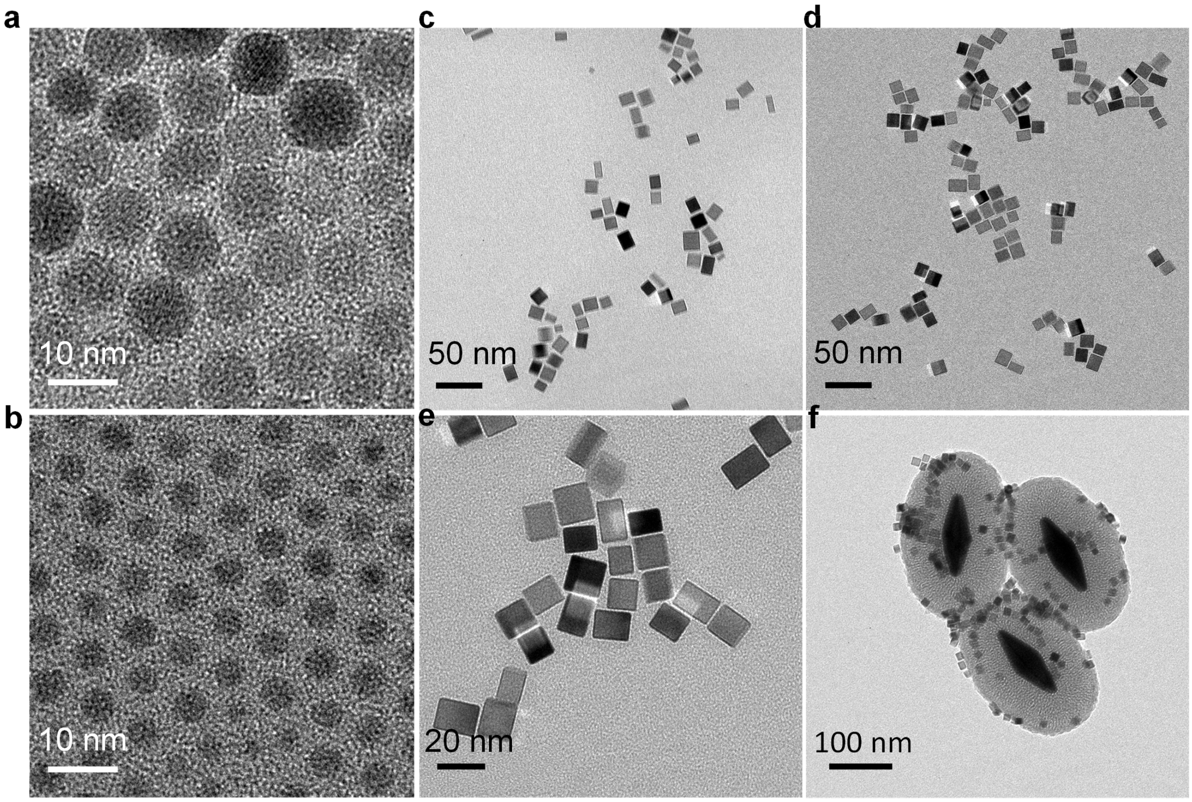


**Supplementary Figure 49** | **a-b,** TEM images of photothermally synthesized (with 15 OD AuBPs measured at 850 nm) **a,** iron oxide nanoparticles (size ≈9 nm, SD 0.5 nm) and **b,** silver nanoparticles (Fig. 4b with 80 °C temperature difference compared to conventional heating using 100 mg silver precursor, size ≈4 nm, SD 0.2 nm). **c-f,** TEM images of palladium nanocubes/bars obtained through **c,** conventional heating (size ≈10-11 nm, SD 0.3 nm) at 85 °C, and **d-e,** photothermal synthesis with 2 OD (measured at 850 nm) AuBPs (size ≈11-12 nm, SD 0.3 nm) at 60 °C with **f,** corresponding Pd@AuBP nanohybrids (size of Pd ≈12-13 nm, SD 0.3 nm).

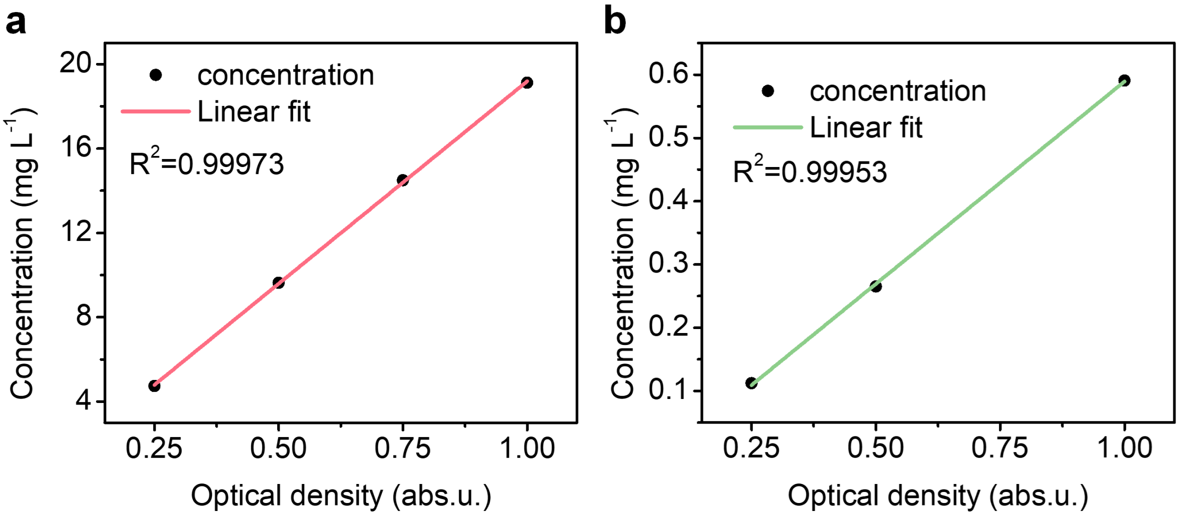


**Supplementary Figure 50** | ICP-OES based correlation between OD (measured at 850 nm) and the concentration of **a,** gold, and **b,** silicon in silica-coated gold bipyramids. For a 1 OD

solution of $SiO_2$@$AuBP_{850}$ (measured at 850 nm), the concentration of Au and Si are 19.2±0.1 ppm and 0.5±0.1 ppm respectively. Source data are provided as a Source Data file.

## 2. Supplementary Tables 1-6.

**Supplementary Table 1** | Effect of photothermal reaction temperature on IONPs formation.

| Precursor (mg) | AuBP (OD measured at 850 nm) | Temperature (°C) | Time (h) | nanoparticle |
|---|---|---|---|---|
| 150 | 15-20 | 200 | 1-4 | monodisperse |
| 150 | 15-20 | 180 | 1-4 | monodisperse |
| 150 | 15-20 | 170 | 1-4 | monodisperse |
| 150 | 15-20 | 160 | 1-4 | nucleation |
| 150 | 15-20 | 150 | 1-4 | - |

**Supplementary Table 2** | Effect of iron precursor concentrations on photothermal nanoparticle formation.

| Precursor (mg) | AuBP (OD measured at 850 nm) | Temperature (°C) | Time (h) | nanoparticle |
|---|---|---|---|---|
| 200 | 15-20 | 200 | 1-4 | aggregated |
| 150 | 15-20 | 200 | 1-4 | monodisperse |
| 100 | 15-20 | 200 | 1-4 | monodisperse |
| 50 | 15-20 | 200 | 1-4 | - |

**Supplementary Table 3** | Effect of photothermal reaction time on IONPs formation.

| Precursor (mg) | AuBP (OD measured at 850 nm) | Temperature (°C) | Time (h) | nanoparticle |
|---|---|---|---|---|
| 150 | 15-20 | 180-200 | 1-2 | monodisperse |
| 150 | 15-20 | 180-200 | 3-4 | monodisperse |
| 150 | 15-20 | 180-200 | 4-6 | aggregated |

**Supplementary Table 4** | Effect of ligand concentrations on nanoparticle formation.

| Precursor (mg), oleylamine (V%) | AuBP (OD measured at 850 nm) | Temperature (°C) | Time (h) | nanoparticle |
|---|---|---|---|---|
| 150 (100%) | 15-20 | 200 | 1-4 | - |
| 150 (80%) | 15-20 | 200 | 1-4 | aggregated |
| 150 (60%) | 15-20 | 200 | 1-4 | monodisperse |
| 150 (40%) | 15-20 | 200 | 1-4 | - |

**Supplementary Table 5** | Effect of regular heating in the presence/absence of AuBPs without light exposure.

| Precursor (mg) | AuBP (OD measured at 850 nm) | Temperature (°C) | Time (h) | nanoparticle |
|---|---|---|---|---|
| 150 | 15-20 | 250 | 1-4 | monodisperse |
| 150 | 15-20 | 210 | 1-4 | monodisperse |
| 150 | 15-20 | 180 | 1-4 | nucleation |
| 150 | 15-20 | 160 | 1-4 | - |
| 150 | 15-20 | 150 | 1-4 | - |

**Supplementary Table 6** | ICP analysis of the intermediate IONP@AuBP nanohybrids isolated at photothermal temperature of 200 °C at different time intervals. For each sample standard deviations were derived from two spectral lines for Au, and five spectral lines for Fe.

| sample | Au 242.795 | Au 267.595 | Au avg. | avg.* | SD | Fe 238.204 | Fe 244.451 | Fe 259.941 | Fe 261.187 | Fe 373.486 | Fe avg. | avg.* | SD | Fe/Au |
|---|---|---|---|---|---|---|---|---|---|---|---|---|---|---|
| | mg $L^{-1}$ | mg $L^{-1}$ | mg $L^{-1}$ | mg $L^{-1}$ | | mg $L^{-1}$ | mg $L^{-1}$ | mg $L^{-1}$ | mg $L^{-1}$ | mg $L^{-1}$ | mg $L^{-1}$ | mg $L^{-1}$ | | |
| **blank** | 0.05 | 0.042 | 0.046 | - | - | 0.174 | 0.117 | 0.159 | 0.145 | 0.137 | 0.146 | - | - | - |
| **45 min** | 1.585 | 1.285 | 1.435 | 1.389 | 0.150 | 0.550 | 0.364 | 0.479 | 0.480 | 0.484 | 0.471 | 0.325 | 0.060 | 0.233 |
| **2h** | 0.728 | 0.611 | 0.669 | 0.623 | 0.058 | 0.232 | 0.171 | 0.21 | 0.195 | 0.187 | 0.199 | 0.052 | 0.020 | 0.084 |
| **3h** | 2.087 | 1.68 | 1.883 | 1.837 | 0.203 | 0.280 | 0.213 | 0.249 | 0.238 | 0.241 | 0.244 | 0.097 | 0.021 | 0.053 |
| **4h** | 0.406 | 0.365 | 0.385 | 0.339 | 0.020 | 0.094 | 0.074 | 0.091 | 0.073 | 0.097 | 0.085 | - | 0.010 | - |

Avg*: average after subtracting the blank.
