# Supplementary material for: Photothermally heated colloidal synthesis of nanoparticles driven by silica-encapsulated plasmonic heat sources
Source: Nat Commun. 2023 Oct 10;14:6355. doi: 10.1038/s41467-023-42167-9 (PMC10564728; doi:10.1038/s41467-023-42167-9)
Supplement: Supplementary file 3 — Description of Additional Supplementary Files [file 41467_2023_42167_MOESM3_ESM.pdf]

Description of Additional Supplementary Files

File Name: Supplementary Movie 1
Description: TEM tomography movie of IONP@AuBP exhibiting the homogenous distribution of iron oxide nanoparticles over the AuBP.
